# Engineering aptamer-based therapeutics for precision cancer immunotherapy: from molecular targeting to clinical applications

**DOI:** 10.3389/fphar.2026.1987479

**Published:** 2026-10-26

**Authors:** Mohammad Asim Azhar, Sana Ahmed, Abdullah Altuhami, Mohammad Shabab, Abdullah M. Assiri, Dieter C. Broering, Tanveer Ahmad Mir

**Affiliations:** 1 Organ Transplant Center of Excellence, King Faisal Hospital and Research Center, Riyadh, Saudi Arabia; 2 Graduate School of Science and Engineering for Education, University of Toyama, Toyama, Japan

**Keywords:** aptamers, cancer immunotherapy, precision oncology, targeted drug delivery, tumor microenvironment

## Abstract

Although cancer immunotherapy has revolutionized cancer therapy, the clinical efficacy of this approach is restricted due to tumor heterogeneity, immune escape, therapeutic resistance, poor tumor penetration, and toxic side effects. Short single-stranded DNA or RNA oligonucleotides, termed aptamers, have high affinity for a given target, low immunogenicity, and high chemical programmability and are emerging as promising agents for precision cancer therapies and immuno-oncology. This review aims to provide a comprehensive, integrated overview of the development of aptamer-based cancer therapeutics, from molecular engineering and pharmacology to clinical applications and translational potential. The discovery and optimization of tumor and immune-specific aptamers have been enhanced by the development of conventional SELEX, Cell-SELEX, *in vivo* SELEX, high-throughput sequencing, microfluidic selection, and AI-assisted selection. To enhance nuclease resistance, circulation time, biodistribution, and tumor accumulation, chemical modifications such as PEGylation, 2′-fluoro, 2′-O-methyl, LNAs, phosphorothioate linkages, circularization, and lipid/albumin binding have been developed. Aptamers can directly regulate oncogenic and immune signaling pathways or be used as targeted delivery vehicles for chemotherapeutic drugs, siRNAs, miRNAs, proteins, peptides, and CRISPR-based systems. Moreover, aptamers targeting tumor-associated biomarkers, cancer stem cells, the tumor vasculature, stromal components, and immune regulatory pathways have the potential to selectively remodel the tumor microenvironment. In addition, they are well-suited to other immunotherapy strategies, including immune checkpoint inhibitors, chemotherapy, radiotherapy, photodynamic or photothermal therapy, oncolytic viruses, and cancer vaccines, thereby further supporting a multifunctional, personalized treatment approach. Although there has been significant progress in pre-clinical research, several issues remain regarding pharmacokinetics, tumor penetration, off-target effects, manufacturing, long-term safety, regulatory approval, and clinical validation. Additional advancements in aptamer engineering, nanotechnology, computational design, and biomarker-directed therapy could potentially enable the development of these platforms into successful next-generation precision cancer immunotherapies.

## 1 Introduction

Cancer is a significant public health burden and continues to be a leading cause of morbidity and mortality, although considerable strides have been made in cancer prevention, diagnosis, and treatment. There is now clear evidence of a significant and changing cancer burden at a global level, particularly from esophageal, gastric, liver, and lung cancer, as well as the increasing burden due to modifiable risk factors (Jin et al., 2025; Li et al., 2025k; Xu Y. et al., 2025). Traditional therapeutic approaches, such as surgery, chemotherapy, radiotherapy, targeted therapy, and hormone therapy, have had a significant impact on survival for many cancers. Still, they can suffer from limitations, including tumor heterogeneity, metastatic spread, acquired drug resistance, and toxic effects (Dagogo-Jack and Shaw, 2018). The hurdles highlight the need for targeted and accurate therapeutic approaches may help to selectively target malignant cells without affecting healthy cells. Cancer immunotherapy has revolutionized cancer therapy by unleashing the host immune system against tumor cells. There is also a growing number of durable clinical responses in certain types of cancers to drugs targeting immune checkpoints such as programmed cell death protein 1 (PD-1), programmed death-ligand 1 (PD-L1), and cytotoxic T-lymphocyte-associated protein 4 (CTLA-4), as well as to adoptive cell therapies and therapeutic cancer vaccines (June and Sadelain, 2018; Ribas and Wolchok, 2018; Braun et al., 2025). Nevertheless, clinical responses to cancer immunotherapy vary: only a fraction of patients achieve long-lasting benefits, and primary or acquired resistance may significantly reduce treatment effectiveness. Various and intertwined factors contribute to this variability, including the composition and activity of the tumor immune microenvironment, insufficient infiltration or activation of effector immune cells, tumor antigen heterogeneity, and molecular mechanisms that enable immune evasion and therapeutic resistance (Fang et al., 2025a; Zhang K. et al., 2026). These restrictions have led to the development of next-generation targeted immunotherapeutic platforms with high molecular specificity and immunomodulatory properties–such as nucleic acid aptamers with excellent target selectivity, low immunogenicity, ease of chemical modification, and versatility for therapeutic payload delivery and immune response modulation. Precision oncology has become a paradigm that has increasingly transformed cancer treatment through molecularly informed patient stratification and treatment selection based on the genomic, transcriptomic, proteomic, epigenetic, and immune profiles of individual tumors (Miglino et al., 2025). Single-cell and spatial profiling, as well as integrated multi-omics analysis, have aided in the identification of more comprehensive genomic alterations, dysregulated signaling networks, molecular subtypes, and predictive or resistance-associated biomarkers (Khaghani et al., 2024). These can reveal clinically relevant heterogeneity and define patient subsets that are likely to respond to targeted or immune-based therapies. Recently published multi-omics studies have identified molecular signatures driving responses to anti-PD-1 or anti-epidermal growth factor receptor (EGFR) therapy and characterized molecular mechanisms of treatment resistance, highlighting the potential of integrated molecular profiling to support biomarker-guided therapeutic stratification (Aung et al., 2025; van der Elst et al., 2026). The advances have led to the development and clinical use of molecularly targeted therapies of oncogenic drivers, including EGFR, human epidermal growth factor receptor 2 (HER2), B-Raf proto-oncogene, anaplastic lymphoma kinase, and Kirsten rat sarcoma viral oncogene homolog (KRAS), resulting in better outcomes for selected molecularly defined patient populations (Yang J. C.-H. et al., 2025; Heymach et al., 2026; Yue et al., 2026). While precision medicine has proven successful, its clinical utility remains limited by intratumoral heterogeneity, clonal evolution, and acquired resistance, underscoring the need for more versatile and multifunctional therapeutic platforms (Alsaed et al., 2025). At the same time, cancer immunology has evolved into a field that has broadened the scope of personalized cancer treatment beyond reliance on genomic alterations alone. New biomarkers, including tumor mutational burden, microsatellite instability, immune cell infiltration, and the composition of the tumor microenvironment (TME), have emerged as useful predictors of immunotherapy response and are increasingly being used in clinical decision-making (Usset et al., 2024). These advances have paved the way to the rapid emergence of targeted immunotherapeutic approaches, featuring the simultaneous recognition of specific molecules and modulation of the immune response (Muquith et al., 2024; Usset et al., 2024). Of these, aptamers have attracted considerable interest for their high binding affinity, outstanding target specificity, low immunogenicity, and amenability to chemical engineering. Aptamers' capacity to specifically bind to tumor-associated biomarkers, deliver therapeutic payloads, and regulate immune signaling pathways makes them promising molecular tools for precision oncology and next-generation cancer immunotherapies (Laowichuwakonnukul et al., 2025; Li et al., 2025i; Tong et al., 2025). Short, single-stranded DNA or RNA oligonucleotides with a structure that can fold into three-dimensional shapes that bind tightly and specifically to a target molecule, similar to the way a monoclonal antibody binds. Aptamers may be synthesized using an algorithm that systematically evolves ligands by exponential enrichment (SELEX) against virtually any target, such as a protein, peptide, small molecule, cell, or even a complex tissue (Chinchilla-Cárdenas et al., 2024; Zhu C. et al., 2024). Aptamers have several pharmacological properties that are advantageous compared to protein-based therapeutics, such as low immunogenicity, high batch-to-batch reproducibility, ease of chemical synthesis, rapid tissue penetration due to their small size, and ability to easily incorporate chemical modifications that can increase stability, circulation time, and target specificity. Aptamers are an extremely versatile platform for therapeutic intervention and molecular targeting due to these properties (Li Y. et al., 2024; Thomas et al., 2024b; Xiao et al., 2024). In oncology, aptamers have also shown great promise as precision-medicine agents that can specifically target tumor-associated antigens (TAAs) and immune regulatory molecules without causing off-target effects. As delivery agents for chemotherapeutic drugs, small interfering RNAs (siRNAs), microRNAs (miRNAs), proteins, nanoparticles, and gene-editing systems, aptamers can enhance therapeutic efficacy and minimize systemic adverse effects (Zhu S. et al., 2024; Ning et al., 2025). In addition, aptamers can be designed to act as modulators of immune checkpoints, to activate co-stimulatory receptors, or to selectively guide immune effector cells to tumor sites, and thus be used in cancer immunotherapy (Ayass et al., 2024; Zhang et al., 2024; Ning et al., 2025). Aptamers have emerged as a promising class of next-generation therapeutics, due to their high target affinity, engineering versatility, and compatibility with combination therapies, making them a key focus in precision cancer treatment. Although significant advances have been made in the development of aptamer technology and cancer immunotherapy, the literature remains largely incoherent, with most review articles focusing on either aptamer engineering and targeted drug delivery or cancer immunotherapy alone, without a unified pharmacological/translational approach to their combination. The therapeutic potential of aptamer-based immuno-oncology has been greatly broadened by recent developments in chemically engineered aptamers, immune checkpoint-targeting aptamers, aptamer–cargo conjugates, nanotechnology-based delivery systems, and combination immunotherapies. Aptamers are intermediate between antibodies and peptides in translation. Aptamers are typically small, single-stranded DNA or RNA molecules, ranging in size from approximately 5–30 kDa. In comparison, conventional IgG antibodies range in size from ∼150 kDa, and many therapeutic peptides in use range from 1 to 5 kDa (Table 1) (Kim et al., 2025b; Su et al., 2025; Nicholas et al., 2026). They are smaller, which can help to penetrate tissue and tumor, but can also lead to rapid renal filtration and hence to shorter systemic exposure (except when chemical modification/molecular conjugation is used). While antibodies tend to have well-known pharmacokinetics and longer circulation times, they are more structurally complex than peptides, penetrate tissues more slowly, and require higher manufacturing demands (Jing et al., 2025). On the other hand, peptides tend to penetrate tissues well, be easily synthesized, have shorter half-lives, and are more quickly subject to proteolytic degradation (Höppner et al., 2025). Chemically synthesized aptamers are typically not immunogenic and can be site-specifically chemically modified, whereas antibodies are biologically produced and may thus cause anti-drug immune responses, depending on their sequence and formulation; peptides are also typically non-immunogenic, but may be immunogenic depending on sequence and formulation (Su et al., 2025; Ren et al., 2026). Aptamers and peptides offer the advantages of chemical synthesis and product uniformity compared to antibody production, which involves the expression, purification, and careful control of protein folding and glycosylation in cells. Therefore, the molecular size, specificity, engineering flexibility, tissue penetration, and manufacturing control of aptamers may be beneficial, the cost advantage may not be shared by all, as chemical modifications, half-life extension strategies, and increasingly complex multivalent and nanomaterial constructs can significantly increase production costs (Table 1) (Gong et al., 2025; Xia et al., 2025; Tao et al., 2026).

**TABLE 1:** Comparative characteristics of aptamers, antibodies, and peptides for therapeutic development.

Parameters	Aptamers	Antibodies	Peptides	References
Typical molecular size	∼5–30 kDa	∼150 kDa for IgG	∼1–5 kDa for many therapeutic peptides	(Bumbaca et al., 2012; Shraim et al., 2022; Wang et al., 2022)
Immunogenicity	Generally low; sequence/modification dependent	Variable; potentially higher, particularly with non-human or engineered sequences	Generally low, but sequence/formulation dependent	(Gao et al., 2013; Puig and Shubow, 2025; Ji et al., 2026)
Manufacturing	Chemical synthesis; defined composition; readily modified	Cell-based biological production; complex purification and quality control	Chemical synthesis; highly scalable for many peptides	(Fields, 2002; Li et al., 2010; Wu et al., 2026)
Pharmacokinetics	Usually short half-life because of renal filtration and nuclease degradation; can be extended by modification/conjugation	Generally prolonged circulation, often days to weeks	Usually short-to-intermediate half-life because of proteolysis and renal clearance	(Ovacik and Lin, 2018; Zhang et al., 2022b; Nordell et al., 2026)
Tissue penetration	Generally favorable because of small size; target and formulation dependent	Generally slower/limited in dense solid tumors because of large size	Generally favorable because of small size; strongly sequence/formulation dependent	(Xiang et al., 2015; Fu and Xiang, 2020; Xiao et al., 2025b)
Cost	Potentially lower for simple constructs; modification and multivalent/nanoparticle designs increase cost	Generally high manufacturing and development complexity	Often relatively economical for chemically synthesized peptides; complex peptides can be costly	(Barman et al., 2023; Chen et al., 2025a; Liu et al., 2026a)
Engineering flexibility	Very high; sequence, chemical modification, multivalency and cargo conjugation	High but generally requires protein engineering	High; sequence and chemical modifications readily introduced	(Fernández-Quintero et al., 2023; Li et al., 2025c; Arshad et al., 2026)

Still, challenges remain in pharmacokinetics, *in vivo* stability, tumor penetration, manufacturing, and clinical translation. Hence, it is timely and necessary to review current knowledge to incorporate recent advances in the molecular design, pharmacological mechanisms, immunomodulatory properties, and clinical applications of aptamer-based precision cancer immunotherapy. This review highlights recent developments in the field of aptamers for pre-clinical and clinical applications, identifies current challenges and opportunities, and proposes a pathway to rationally develop next-generation aptamer therapeutics for translation into effective, personalized cancer therapies.

## 2 Molecular basis of aptamer therapeutics

Aptamers' therapeutic potential largely depends on their distinct molecular architecture, physicochemical properties, and target-recognition capabilities. Aptamers act by binding molecular targets through highly specific interactions that depend on the aptamer’s tertiary structure and nucleotide sequence (Guérin et al., 2025; Yang Z. et al., 2025). Aptamers work by interacting with the molecular target through highly specific tertiary-structure- and nucleotide-sequence-mediated interactions, unlike conventional small-molecule drugs and protein-based biologics (Gauger et al., 2025). They have been shown to selectively recognize a wide range of biologically relevant targets, including proteins, peptides, carbohydrates, whole living cells, and extracellular vesicles, making them attractive and versatile tools for targeted therapy, molecular imaging, and drug delivery (Elsharkasy et al., 2025). Moreover, the engineering and chemical modification of aptamers have made significant progress, with enhanced stability, pharmacokinetic properties, and therapeutic potential, rendering them promising tools in precision cancer immunotherapy (Zhou Y. et al., 2025; Ren et al., 2026). Therefore, it is critical to understand the molecular basis of aptamer therapeutics to appreciate their mechanism of action and rationally design next-generation aptamers.

### 2.1 Structure and properties of aptamers

#### 2.1.1 DNA vs. RNA aptamers

DNA and RNA aptamers are distinct, with different structural and functional properties that affect their therapeutic potential. In general, DNA aptamers have superior chemical stability, resistance to nuclease degradation, ease of synthesis, and lower production costs, making them suitable for diagnostic use and systemic delivery of therapeutics (Daum et al., 2024; Li et al., 2024a). Unlike DNA, RNA aptamers are more conformationally flexible because of the presence of the 2′-hydroxyl group on ribose, which enables them to assume more complex tertiary structures that frequently yield higher binding affinity and target specificity. In physiologic conditions, however, native RNA aptamers degrade quickly, requiring chemical modification of the structure, for example, via 2′-fluoro (2′-F), 2′-O-methyl (2′-OMe), locked nucleic acid (LNA), or phosphorothioate substitutions to ensure stability and circulation time (Gauger et al., 2025; Zhang Y. et al., 2025). Hence, the choice between DNA and RNA aptamers depends on the therapeutic use, the nature of the target, and the desired pharmacological attributes.

The biological activity of aptamers is determined by their ability to fold into a well-defined 3D structure that can form highly specific interfaces for binding to the target. Post-selection, intramolecular base pairing yields a variety of secondary structures, such as stem-loops, hairpins, internal bulges, pseudoknots, and G-quadruplexes, which then form stable tertiary structures (Climaco et al., 2025; Lau et al., 2025). These complex architectures can create complementary surfaces that recognize target molecules through hydrogen-bonding, electrostatic, van der Waals, hydrophobic, and π–π stacking interactions (Wang Y.-P. et al., 2024; Ochoa and Milam, 2025). Importantly, however, even subtle changes in the nucleotide sequence or in environmental conditions, such as ionic strength, pH, and temperature, may affect aptamer folding and, consequently, binding performance. Optimization of the structure through rational design has thus; emerged as a core strategy for improving the stability, specificity, and therapeutic applications of aptamers (Yu H. et al., 2025; Muniz et al., 2026).

What sets aptamers apart as precision therapeutic agents is their high binding affinity and excellent target specificity, which remain subjects of research interest. The equilibrium dissociation constant (K_D_) for aptamer–target binding is generally in the picomolar to nanomolar range, comparable to that of high-affinity monoclonal antibodies (Soxpollard et al., 2024; Alkhamis et al., 2025). The very high level of molecular recognition, due to the exact fit between the folded aptamer structure and the target’s epitope, allows the aptamer to distinguish among highly similar proteins, different receptor conformations, mutant and wild-type versions of a protein, and even single amino acid changes (Quintela et al., 2025; Yan et al., 2025). The specificity helps inhibit off-target interactions and greatly reduces the likelihood of systemic toxicity, making aptamers especially well-suited for targeted cancer therapy and immunotherapy. Further, the development of aptamers with improved pharmacological performance and clinical translational potential has been boosted by advances in SELEX methodologies, computational modeling, and structural optimization (Zhang Y. et al., 2025).

### 2.2 SELEX technologies

#### 2.2.1 Conventional SELEX

The basic method for aptamer discovery is called SELEX, which entails a series of binding and partitioning rounds, amplification, and enrichment, to obtain high-affinity binding aptamers to a purified molecular target (Yu H. et al., 2025; Zhang Y. et al., 2025). The first step is the creation of a very complex library of nucleic acids - usually 10^13^–10^15^ - and then mixing this library with the target molecule. Unbound oligonucleotides are separated from bound sequences, which are amplified either by PCR (for DNA aptamers) or reverse transcription-PCR (for RNA aptamers) and subjected to subsequent selection cycles to obtain highly bound sequences (Guérin et al., 2025; Wu Z. et al., 2025). While SELEX has been used in the past to generate aptamers against many proteins and small molecules, using purified targets may not capture the true native conformation or post-translational modifications, and the iterative selection process can be time-consuming and labor-intensive (Yang X. et al., 2025; Zhang Y. et al., 2025). However, it remains one of the most popular methods for aptamer selection and has been used to develop next-generation aptamer SELEX platforms (Figure 1).

**FIGURE 1:**
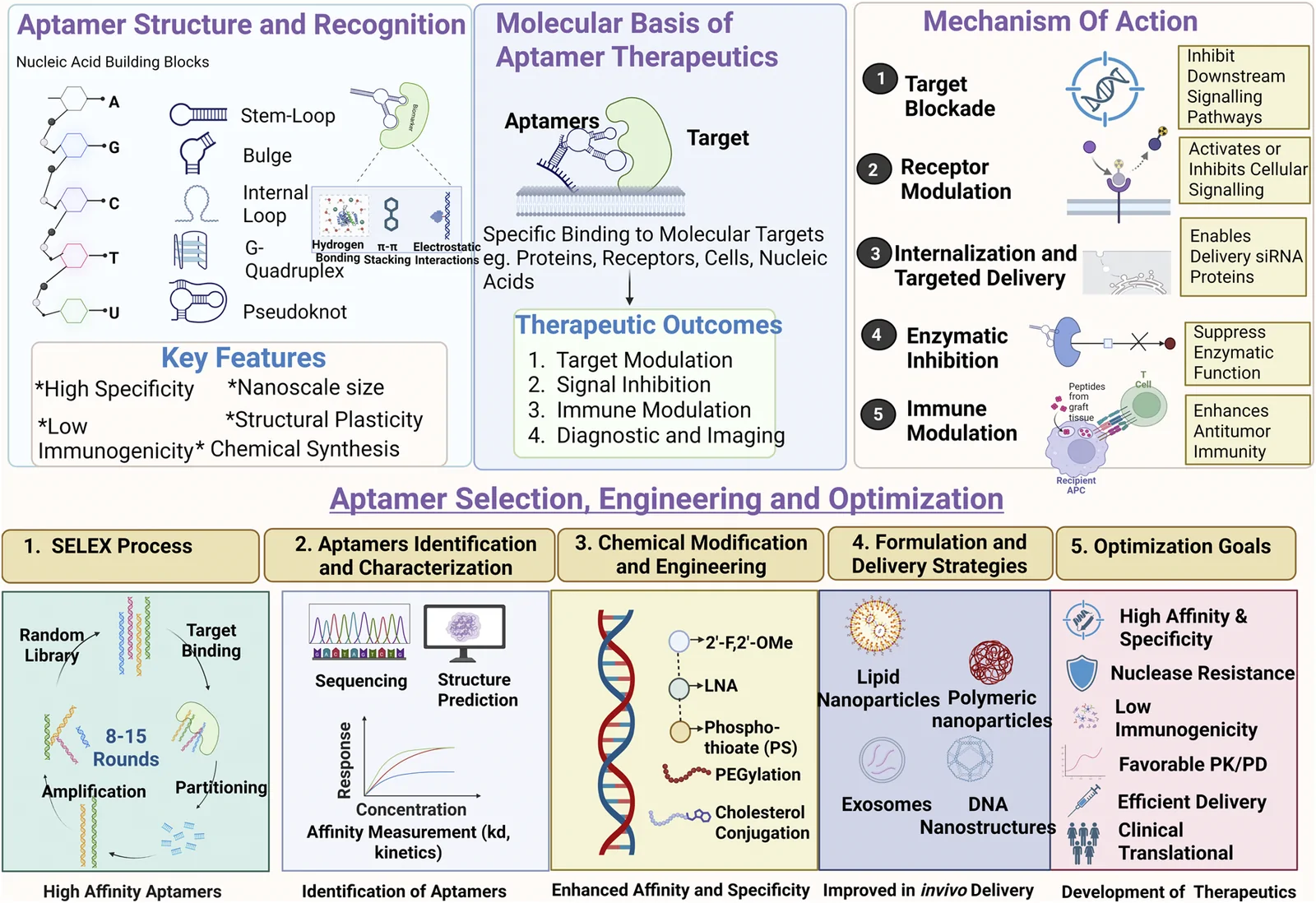
Molecular basis of aptamer therapeutics: structural organization, mechanisms of action, and engineering strategies.

#### 2.2.2 Cell-SELEX

Cell-SELEX is designed to overcome the pitfall of using purified targets as the selection substrate by using intact living cells to preserve the native protein conformation, membrane organization, and physiological receptor expression (Kwon et al., 2025; Li W. et al., 2025). This can be used to identify aptamers against cell-surface biomarkers without prior knowledge of the molecule, thereby greatly advancing the discovery and targeting of cancer-specific biomarkers. Using non-target or healthy cells for negative selection increases specificity by removing sequences that bind to common cellular components. The use of cell-SELEX has led to the development of aptamers for clinically important cancer biomarkers, including epithelial cell adhesion molecule (EpCAM), HER2, EGFR, mucin-1 (MUC1), prostate-specific membrane antigen (PSMA), and CD44, which can be used in tumor imaging, targeted drug delivery, and precision immunotherapy (Kim et al., 2025a; Liu K. et al., 2025). Since aptamers are selected in biologically relevant conditions, the performance of the aptamers produced by cell-SELEX is often superior to that of the aptamers from a conventional SELEX (Bakhtiari et al., 2025; Kwon et al., 2025).

#### 2.2.3 *In vivo* SELEX

*In vivo* SELEX is a significant improvement over aptamer selection methods, in which the evolutionary process occurs in living animal models rather than *in vitro* (Korhonen et al., 2024; Doherty et al., 2025). After systemic injection of a diversified aptamer library, the sequences selectively captured in the target tissue, tumor, or pathological microenvironment are recovered, amplified, and reselected in subsequent rounds. This approach enables the selection of aptamers with high target affinity, favorable pharmacokinetic properties, long circulation times, tissue-penetrating properties, and biological stability, thereby increasing their translational relevance (Li C. et al., 2025; Liu K. et al., 2025). In oncology, *in vivo* SELEX has been used to identify aptamers that selectively target tumors, can cross biological barriers, and can recognize targets in the complex TME. *In vivo* SELEX is a powerful platform for the discovery of clinically relevant aptamers with greater therapeutic potential, albeit more challenging and resource-intensive than conventional methods (Korhonen et al., 2024; Li C. et al., 2025).

#### 2.2.4 Microfluidic and AI-assisted SELEX

In recent years, the discovery of aptamers has become significantly more efficient and accurate due to innovations in microfluidics and artificial intelligence (AI) (Yang X. et al., 2025). Microfluidic SELEX combines selection, washing, partitioning, and amplification on miniaturized platforms, thereby reducing reagent consumption, selection time, and experimental bias, and enabling automated high-throughput screening (HTS) (Zhang S. et al., 2025; Shao et al., 2026). These may be complemented by advances in AI and machine learning algorithms that can analyze large-scale sequencing datasets to identify enriched sequence motifs, predict secondary and tertiary structures, estimate aptamer binding affinity, and prioritize high-performance aptamer candidates before experimental testing. The number of experimental selection cycles can be reduced by using deep learning models to optimize nucleotide sequences, enhance structural stability, and predict aptamer–target interactions. Microfluidics combined with AI-driven computational design is now revolutionizing aptamer engineering, making it faster, more cost-effective, and more data-driven, thereby driving the development of next-generation therapeutics for precision oncology and cancer immunotherapy (Thi Tran et al., 2025; Zhao Z. et al., 2025).

#### 2.2.5 HTS in aptamer discovery

The use of HTS, or next-generation sequencing, has completely transformed the aptamer discovery process by enabling comprehensive analysis of millions of sequences generated during SELEX (Shareef and Hariprasad, 2025; Drees et al., 2026). HTS can provide a detailed view of sequence diversity, candidate enrichment trends, and evolutionary selection across successive SELEX rounds. In contrast, conventional cloning and Sanger sequencing characterize only a limited number of enriched candidates (Yu H. et al., 2025). HTS, in association with bioinformatics analyses, allows for the identification of enriched sequence families, conserved binding motifs, mutational patterns, and structural features that correlate with high-affinity target recognition. This method enables the identification of good aptamer candidates, reduces the number of selection cycles, and enhances the accuracy and repeatability of aptamer identification. With the ongoing development of sequencing techniques and computational analysis, HTS has become an essential part of the 21st-century aptamer discovery process, enabling the rational design of highly targeted therapeutic aptamers with greater translational potential (Zhao Z. et al., 2025).

### 2.3 Chemical engineering for therapeutic stability

#### 2.3.1 PEGylation

PEGylation is a widely used method for enhancing the pharmacokinetic parameters of therapeutic aptamers by covalently attaching a polyethylene glycol chain (usually to the 5′ or 3′ end of the oligonucleotide) (Table 2) (Haruta et al., 2017; Sun et al., 2025). Unmodified aptamers are typically too small to filter through the kidney and hence have a very short plasma half-life. The PEGylated aptamers have enhanced hydrodynamic size, which reduces glomerular filtration, extends systemic circulation, improves their aqueous solubility, and decreases nonspecific protein interactions (Liu et al., 2024). This modification has been successfully implemented in various clinically tested aptamers, such as pegaptanib, pegnivacogin, and olaptesed pegol (Steurer et al., 2019). While PEG antibodies have been observed in individual patients, PEGylation is an extremely promising strategy for increasing the biological half-life and therapeutic activity of aptamer-based therapeutics (Table 2) (Moreno et al., 2019; Lin et al., 2023).

**TABLE 2:** Pharmacokinetic optimization strategies for therapeutic aptamers.

Strategy	Mechanism of action	Primary pharmacokinetic benefit	Potential limitation	References
PEGylation	Conjugation of PEG increases hydrodynamic size	Reduces renal filtration and prolongs plasma half-life	Pre-existing or treatment-induced anti-PEG antibodies may promote accelerated clearance, complement activation, or hypersensitivity reactions	(Gao et al., 2024)
2′-F modification	Ribose substitution enhances resistance to RNase degradation	Improves serum stability while maintaining RNA structure	Sequence-dependent effects on aptamer folding	(Kratschmer and Levy, 2017)
2′-OMe modification	Replaces ribose 2′-OH with methyl group	Increases nuclease resistance and reduces innate immune activation	Excessive modification may reduce binding affinity	(Maio et al., 2017; Alharbi et al., 2026)
LNA	Constrains ribose conformation to stabilize duplex structure	Enhances thermal stability, target affinity, and nuclease resistance	Excessive incorporation may alter tertiary structure; innate immune effects can be sequence- and position-dependent	(Jørgensen et al., 2014)
Phosphorothioate backbone modification	Sulfur substitution in phosphate backbone reduces nuclease susceptibility	Improves metabolic stability and circulation time	Increased nonspecific protein binding and context-dependent TLR9/complement activation	(Ebrahimi et al., 2025)
Circular aptamers	Covalent linkage of 5′ and 3′ termini eliminates exonuclease-sensitive ends	Greatly enhances serum stability and prolongs biological activity	More complex synthesis and structural optimization	(Liu et al., 2025e)
Spiegelmers	Use of mirror-image L-nucleotides prevents nuclease recognition	Exceptional *in vivo* stability and prolonged persistence	Very low adaptive immunogenicity; innate responses may remain sequence- and delivery-dependent	(Yu et al., 2025a)
Lipid conjugation	Attachment of lipophilic moieties promotes plasma protein association	Extends circulation and enhances cellular uptake	May alter biodistribution and increase formulation complexity	(Pan et al., 2026)
Albumin-binding strategy	Direct albumin conjugation or incorporation of albumin-binding motifs	Exploits albumin recycling to prolong systemic exposure	Additional engineering required to preserve target binding	(Yang et al., 2025e)
Nanoparticle encapsulation/conjugation	Incorporation into liposomes, polymeric nanoparticles, or lipid nanoparticles	Protects against degradation, enhances tumor accumulation, and enables controlled release	Increased manufacturing complexity and regulatory considerations	(Sarkar et al., 2024)

#### 2.3.2 LNAs

Conformationally constrained nucleotide analogs are LNAs in which the 2′-oxygen and 4′-carbon of the ribose ring are linked by a methylene bridge, which fixes the sugar in a rigid C3′-endo conformation (Figure 1) (Petersen et al., 2000; Koshkin et al., 2001). This structural constraint has a significant effect on the duplex thermal stability, nuclease resistance, and target binding affinity, with very little impact on the overall architecture of the aptamer. Insertion of LNA residues at strategically important sites improves the rigidity of the structure and the active conformation of the aptamer, sometimes even improving binding kinetics and serum stability (Schmidt, 2004; Jørgensen et al., 2014). If too many LNA substitutions or substitutions in the wrong positions are used, the LNA may not fold into its tertiary structure, and the recognition of the target molecule may be affected, so that sequence-specific optimization must be performed. Partially modified LNAs are therefore frequently used to prepare highly stable therapeutic aptamers and potentially in combination with other ribose modifications (Table 2) (Shi et al., 2014).

#### 2.3.3 2′-F and 2′-OMe modifications

The two most widely used ribose modifications are 2′-F and 2′-OMe, which improve the biostability of RNA aptamers (Table 2). The changes involve replacing the native 2′-hydroxyl group of ribose while retaining the structural integrity necessary for target recognition, thereby limiting RNase-mediated hydrolysis of the molecule (Maio et al., 2017; Odeh et al., 2019). The 2′-F modification also significantly enhances the nuclease resistance of the RNA, and stabilizes secondary structures such as G-quadruplexes; 2′-OMe also contributes to thermal stability, reduces immune recognition, and extends serum half-life (Maio et al., 2017). These modifications are often added in or at the end of SELEX and are commonly employed in combination with PEGylation, phosphorothioate linkages, or LNA residues to gain synergistic improvements in pharmacokinetics and therapeutic durability (Figure 1). The use in clinically evaluated aptamers highlights their role as the hallmark modifications for next-generation aptamer therapeutics (Table 2) (Kratschmer and Levy, 2017).

#### 2.3.4 Circular aptamers

Linear aptamers are covalently coupled at both 5′ and 3′ ends to create circular aptamers that do not possess free ends that could be targeted by exonuclease degradation. Circularization can significantly enhance resistance to nucleases, structural rigidity, and the stability of the bioactive conformation, thereby increasing serum stability and functional activity (Mao et al., 2020; Yang et al., 2023). Because of the reduced conformational flexibility, in many cases, circular aptamers have higher target affinities and better biological activity than linear aptamers. Enzymatic ligation, click chemistry, and other bioorthogonal cyclization strategies have recently been developed to enable the efficient generation of circular aptamers, which may become a more attractive platform for targeted drug delivery, molecular imaging, and cancer immunotherapy applications that require long *in vivo* activity (Wang J. et al., 2025).

#### 2.3.5 Spiegelmers

Spiegelmers are aptamers composed entirely of L-nucleotides (which don't occur in DNA or RNA), not D-nucleotides (Eulberg and Klussmann, 2003). Spiegelmers are highly resistant to enzymatic degradation because they are not recognized by the endogenous nucleases, which recognize only D-configured nucleic acids, and are also highly stable *in vivo* (Olea et al., 2015). They must have developed using a special “selection-reflection” approach, in which conventional SELEX is first performed against the mirror image of the natural sequence, followed by chemical synthesis to obtain the corresponding L-aptamer that binds to the natural sequence (Chen et al., 2022). In general, spiegelmers are poorly immunogenic, as their L-nucleotide structure is poorly recognized by the usual pathways for nucleic acid degradation and by immune recognition pathways; however, it is not entirely possible to rule out sequence- and delivery-dependent innate immune responses. This unique class of aptamer therapeutics has now advanced to clinical trials, including several PEGylated spiegelmers targeting CXCL12 and hepcidin (Table 2) (Stinnakre and Van Renterghem, 1986; Van Eijk et al., 2013).

#### 2.3.6 Pharmacokinetic optimization

The rapid renal clearance, poor serum stability, and nuclease degradation are important hurdles that can limit the therapeutic potential of native aptamers, and this is known as pharmacokinetic optimization in aptamer engineering. To overcome these drawbacks, several strategies for chemical and structural modification have been developed to enhance absorption, distribution, metabolism, and excretion without compromising target binding or activity. Typical methods involve modifying the backbone to be less susceptible to enzymatic degradation (e.g., phosphorothioate backbone), incorporation of LNAs to increase the structural stability, long-circulating PEGylation to increase the hydrodynamic size and circulation half-life, long half-life in the tumor site by multivalent or nanoparticle-based formulation, and exonuclease-resistant termini (e.g., by circularization) (Vater et al., 2013; Hoellenriegel et al., 2014). Recently, other albumin-binding ligands, lipid conjugation, and biomimetic nanocarriers have been investigated to enhance biodistribution and tissue-specific delivery further (Yang S. et al., 2025). Together, these pharmacokinetic optimization strategies greatly improve plasma half-life, enhance tumor targeting, reduce dosing frequency, and increase the translatability of aptamer-based therapeutics for precision cancer immunotherapy (Sun et al., 2025).

Importantly, pharmacokinetic optimization can also result in immunologic risks associated with the modification, which can be weighed alongside its intended effects. The anti-PEG antibody response is not a direct activation of nucleic acid-sensing pathways; instead, pre-existing anti-PEG antibodies can be induced by PEG-containing therapeutics, leading to complement activation, accelerated blood clearance, and hypersensitivity reactions, especially upon repeat exposure (Zheng et al., 2025; Chen et al., 2026a). The phosphorothioate backbones make the strands resistant to nucleases, enhance protein association, and may exhibit enhanced non-specific protein binding, depending on sequence, phosphorothioate content, and cellular context. Phosphorothioate backbones may activate the innate immune response through several mechanisms, including TLR9 signaling (Croft et al., 2024; Pytte et al., 2026). Recent research has demonstrated that TLR9 activation requires specific sequences and modification positions; some LNA substitutions may actually increase or decrease TLR9 activation (Riera-Tur et al., 2024). By contrast, spiegelmers with L-nucleotides are poorly immunogenic in the adaptive immune system, which is attributed to their mirror-image configuration and resistance to recognition by the usual nucleases, but not to the activation of innate immunity by some G-rich L-RNA sequences after delivery into the cytoplasm (Yu C.-H. et al., 2025). In addition to nucleotide chemistry and sequence, the backbone chemistry, higher-order structure, dose, route of administration, and cellular localization all play a role in determining the immunological profile of a modified aptamer, not just the modification itself (Ren et al., 2026).

Main pharmacokinetic optimization strategies used to enhance the *in vivo* properties of therapeutic aptamers. All these approaches increase serum stability, prolong systemic circulation, decrease renal clearance, and enhance tumor-specific delivery with high target affinity and therapeutic efficacy. The use of aptamer-based therapeutics is becoming more widespread, with complementary strategies such as ribose modifications, PEGylation, and nanoparticle-based delivery.

Aptamers may fold into unique secondary and tertiary structures (such as stem-loops, hairpins, bulges, pseudoknots, and G-quadruplexes) that enable high-affinity and high-specificity recognition of a wide range of molecular targets through hydrogen bonding, electrostatic interactions, π–π stacking, and van der Waals forces (Jiang Y. et al., 2025). After binding to the target, aptamers have various therapeutic properties such as receptor antagonism or agonism, the inhibition of the interaction between the receptor and its ligand, modulation of the intracellular signaling pathways, receptor-mediated endocytosis for targeted delivery of therapeutic cargoes (drugs, siRNAs, miRNAs, proteins, etc., CRISPR components), enzymatic inhibition, and immune modulation (Xue et al., 2024; Ning et al., 2025). Discovery and optimization of therapeutic aptamers include iterative SELEX-based selection, HTS, chemical modification (e.g., 2′-fluoro, 2′-O-methyl, LNAs, phosphorothioate linkages, cholesterol conjugation, and circularization), and advanced delivery platforms (e.g., lipid nanoparticles, polymeric nanoparticles, exosomes, and DNA nanostructures) (Figure 1) (Shareef and Hariprasad, 2025; Zhang Y. et al., 2025). Taken together, all of these engineering approaches enhance the binding affinity, nuclease resistance, pharmacokinetic properties, tumor-targeting efficiency, and clinical translatability of next-generation aptamer-based therapeutics for precision oncology and cancer immunotherapy.

## 3 Pharmacological principles of aptamer-based cancer therapy

The future of therapeutic applications of aptamers in clinical trials relies not only on the high specificity of the targeted molecules but also on detailed knowledge of the pharmacological principles governing their biological activity, *in vivo* behavior, and safety profile. As aptamers are nucleic acid-based agents that differ markedly from small-molecule drugs and monoclonal antibodies in their pharmacological properties, they require careful consideration of their mechanisms of action, pharmacokinetic properties, and potential toxicities (Ding et al., 2023). Pharmacodynamically, aptamers can act by targeting a specific protein, binding to a receptor, and modulating signaling pathways within the cell, thereby influencing tumor progression and immune regulation (Wei Y. et al., 2024). Pharmacokinetic properties of these also affect their clinical performance, including absorption, biodistribution, metabolism, tissue penetration, and elimination, which can be optimized through rational chemical modifications and advanced delivery systems. Safety and toxicity, including immunogenicity, off-target effects, susceptibility to nucleases, and long-term tolerability, are also crucial considerations. These three interrelated pharmacological parameters—pharmacodynamics, pharmacokinetics, and safety and toxicity—have been discussed to help elucidate the factors that contribute to the efficacy, translational potential, and clinical success of aptamer-based therapeutic agents (Sun et al., 2025; Ren et al., 2026).

### 3.1 Pharmacodynamics

#### 3.1.1 Target recognition

The basic pharmacodynamic property of aptamers is target recognition, which is closely related to therapeutic specificity in cancer treatment. Aptamers bind to their targets via sequence-dependent 3D conformations that create highly complementary binding interfaces, enabling binding to proteins, glycoproteins, receptors, enzymes, extracellular matrix (ECM) components, and whole cells, with K_D_ values in the pico to-nanomolar range (Chen et al., 2024c; Alkhamis et al., 2025). The small size of aptamers allows them to bind with minimal steric hindrance to small-molecule targets, conformationally dynamic proteins, cryptic epitopes, and other targets, unlike antibodies. Aptamers have been developed to TAAs such as EGFR, HER2, EpCAM, PSMA, nucleolin, MUC1, and PD-L1, thereby selectively intervening in tumor therapy without affecting healthy cells (Guo et al., 2025; Su et al., 2025). This is their use as targeted drug carriers and immune-modulating agents in precision oncology, as well as their direct use as therapeutics, where precise molecular recognition is key.

#### 3.1.2 Receptor antagonism/agonism

In addition to target binding, aptamers also have pharmacological functions as receptor antagonists or agonists, regulating signaling pathways associated with tumor growth and immune responses. Antagonistic aptamers act by competitively blocking ligand–receptor interactions and/or receptor activation, thereby inhibiting downstream oncogenic signaling and immune evasion (Wei Y. et al., 2024; Wu et al., 2024). Examples of such notable compounds include aptamers that target PD-1, PD-L1, VEGF, and CXCL12, all of which block immune checkpoint signaling pathways or the effects of angiogenesis or chemokine-driven tumor progression (Luo et al., 2023; Giordano et al., 2024). In contrast, agonistic aptamers are generally designed as multivalent molecules that can cluster and activate the receptors, mimicking the action of natural ligands (Cong et al., 2024; Wakano et al., 2025). Agonistic aptamers against co-stimulatory receptors (such as 4-1BB, CD137; OX40, CD134; CD28) have been shown to stimulate T cell activation, proliferation, cytokine production, and tumor immunity. The ability to rationally design antagonists or agonists offers unparalleled versatility in pharmacological applications and justifies the use of aptamers for targeted cancer therapy and immunotherapy (McNamara et al., 2008; Pratico et al., 2013).

#### 3.1.3 Signal transduction modulation

Aptamers can regulate intracellular signaling by selectively blocking receptor activation and/or ligand binding, thereby modulating downstream molecular pathways involved in tumor cell proliferation, survival, metastasis, angiogenesis, and immune function. Aptamers can inhibit onco-cytokines and oncogenic pathways, including PI3K/AKT/mTOR, RAS/RAF/MEK/ERK (MAPK), JAK/STAT, NF-κB, and Wnt/β-catenin, thereby decreasing cell proliferation, increasing apoptosis, and inhibiting tumor invasion (Wei Y. et al., 2024; Dwivedy et al., 2025; Li et al., 2025g). Aptamers also modulate immune signaling pathways in cancer immunotherapy, such as by inhibiting inhibitory checkpoint pathways (e.g., PD-1/PD-L1 and CTLA-4) or activating co-stimulatory pathways (e.g., OX40 and 4-1BB), thereby promoting cytotoxic T cell and NK cell activation. In addition, aptamers can carry functional cargo such as siRNAs, miRNAs, antisense oligonucleotides, and CRISPR/Cas components, thereby enabling simultaneous modulation of intracellular gene expression and signaling networks (Luo et al., 2023; Sarkar et al., 2024; Jiang T. et al., 2025). The ability to fine-tune multiple pathways exemplifies the pharmacodynamic flexibility of aptamers and underscores their growing relevance in next-generation precision cancer therapy.

### 3.2 Pharmacokinetics

#### 3.2.1 Distribution

Systemic distribution of aptamers is influenced mostly by their molecular size, charge, conformational structure, plasma protein binding, and chemical modifications. Aptamers are relatively small molecules (5–30 kDa) that easily extravasate from the blood vessel and are easily diffused into tissues (Table 3) (Sun et al., 2025; Yang S. et al., 2025). Their distribution is, however, limited by rapid renal clearance and enzymatic degradation, which can be overcome by chemical modification, such as PEGylation, lipid conjugation, or albumin binding (Fallah et al., 2024). Non-specific tissue accumulation is typically low with aptamers owing to their high target specificity, which provides good target-to-background ratios. Ultimately, the distribution in tissues is determined by the density of target receptor expression, vascular permeability, and the physicochemical parameters of the aptamer, which may affect therapeutic efficacy in solid and hematological malignancies (Table 3) (Li Y. et al., 2024; Park et al., 2024).

**TABLE 3:** Key pharmacokinetic parameters influencing the therapeutic performance of aptamer-based cancer therapeutics.

Pharmacokinetic parameter	Primary determinants	Impact on therapeutic performance	Representative optimization strategies	References
Distribution	Molecular size, charge, structural conformation, plasma protein binding, chemical modifications	Determines tissue exposure and target accessibility	PEGylation, albumin-binding motifs, lipid conjugation	(Park et al., 2023a; Li et al., 2024c)
Tumor penetration	Aptamer size, vascular permeability, ECM density, interstitial fluid pressure, target accessibility	Influences intratumoral diffusion and homogeneous target engagement	Nanocarriers, multivalent aptamers, ECM-modulating strategies, stimuli-responsive delivery systems	(Wang et al., 2024a; Liu et al., 2025c)
Clearance	Renal filtration, nuclease degradation, molecular weight, structural stability	Controls plasma half-life, systemic exposure, and dosing frequency	PEGylation, 2′-F/2′-OMe modifications, LNA incorporation, phosphorothioate backbone, circularization	(Ding et al., 2023; Pan et al., 2026)
Biodistribution	Target expression, administration route, delivery platform, molecular size, chemical modifications	Determines tissue selectivity, tumor accumulation, and off-target exposure	Target-specific engineering, nanoparticle-based delivery, molecular imaging-guided optimization	(Park et al., 2023a; Ren et al., 2025)

#### 3.2.2 Tumor penetration

Aptamer-based cancer therapeutics are often successful if they can efficiently penetrate the tumor. Given their size, aptamers can enter the perivascular region of a solid tumor to access tumor cells beyond it. They are rapidly distributed throughout the tumor interstitium to achieve uniform target engagement, especially in tumors with dense cellular architecture (Table 3) (Xiang et al., 2015; Li Y. et al., 2024). However, it is still possible to limit penetration due to increased interstitial fluid pressure, abnormal tumor vasculature, ECM deposition, hypoxic areas, and stromal barriers within the TME. Current strategies overcome these drawbacks by engineering multivalent aptamers, stimuli-responsive nanocarriers, ECM-modulating systems, and nanoparticle-based formulations to improve intratumoral retention and therapeutic delivery without compromising targeted specificity (Ma et al., 2024; Liu et al., 2025c).

#### 3.2.3 Clearance

Therapeutic aptamers are primarily cleared from the blood by renal filtration, as their molecular weight is typically below the glomerular filtration threshold. As a result, unmodified aptamers typically have plasma half-lives of just minutes to a few hours, which is too short a therapeutic window (Table 3). Metabolic degradation is facilitated by nucleases circulating in the bloodstream, especially for unmodified RNA aptamers, and is also involved in renal elimination (Alsenousy et al., 2025; Ren et al., 2026). Several chemical modifications, such as PEGylation, use of 2′-F and 2′-OMe nucleotides, LNAs, phosphorothioate linkages, circularization, and albumin binding, have been developed to lower renal clearance and increase nuclease resistance, thereby increasing the systemic persistence of the modified nucleosides (Liu Z. et al., 2025; Yang S. et al., 2025). These pharmacokinetic optimization strategies dramatically extend circulation time, increase exposure to tumors, and can reduce dosing frequency, thereby improving the therapeutic utility of aptamer therapeutics (Table 3).

#### 3.2.4 Biodistribution

Biodistribution refers to the time- and tissue-specific localization of aptamers after administration, a key factor in determining their efficacy and safety in therapy. Aptamers are highly specific; they tend to localize to tissues that express their cognate targets and exhibit low levels of nonspecific uptake in healthy organs (Park S. M. et al., 2023; Korhonen et al., 2024). However, there are often measurable accumulations in the liver and kidneys due to physiological clearance mechanisms. Several factors affect the biodistribution of aptamers, including the aptamer sequence, molecular size, chemical modifications, route of administration, target availability, and delivery platform (Ding et al., 2023). Current *in vivo* imaging techniques, including positron emission tomography or single-photon emission computed tomography, fluorescence imaging, and magnetic resonance imaging, are increasingly used to monitor *in vivo* distribution, evaluate target engagement, and support the rational optimization of aptamer-based therapeutics for precision oncology (Table 3) (Kong et al., 2024).

Chemical engineering and advanced delivery platforms, tailored for rational optimization of distribution, tumor penetration, clearance, and biodistribution, enhance systemic stability, improve tumor targeting, minimize off-target accumulation, and ultimately increase the therapeutic efficacy of aptamer-based precision cancer therapies.

### 3.3 Safety and toxicity

#### 3.3.1 Immunogenicity

Unlike protein-based drugs, aptamers have a low inherent immunogenicity, with minimal induction of anti-drug antibody production and adaptive immune responses. Aptamers lack an Fc region, which makes them less likely to activate complement pathways or Fc receptor-mediated immune functions than monoclonal antibodies (Al-Youssef et al., 2016; Duan et al., 2024). The immunologic effects of chemical modification, however, are inconsistent and influenced by the nucleotide chemistry, sequence, extent of modification, backbone structure, and intracellular localization of the modified nucleoside. Unmodified or partially modified nucleic acid aptamers with immunostimulatory motifs, such as CpG-rich sequences, may activate endosomal TLR3, TLR7, TLR8, and TLR9, thereby triggering type I interferons and pro-inflammatory cytokines (Tu et al., 2020; Farooq et al., 2026). Chemical modifications (e.g., 2′-F, 2′-OMe) can reduce recognition by multiple nucleic acid-sensing pathways, while LNA and phosphorothioate modifications may have variable effects and cannot be considered a universal suppressor (Bishani et al., 2024). Phosphorothioate linkages can enhance non-specific protein binding and, depending on sequence and cellular environment, may result in innate immune activation. LNA incorporation can likewise have sequence- and position-dependent effects on TLR9 recognition (Wang Y. et al., 2026). Renal clearance is typically decreased, and there are unique immunological concerns, as pre-existing or antibody-mediated (to either PEG or the PEGylated molecule) may cause accelerated blood clearance, complement activation, or immune-hypersensitivity reactions (Zheng et al., 2025; 2026). Spiegelmers are usually poorly immunogenic, due to their L-nucleotide structure, but there is no absolute guarantee that there are no innate inflammatory responses induced by the sequence and delivery of a spiegelmer (Yu and Sczepanski, 2023). Thus, immune-toxicological studies may focus on the modified aptamer construct rather than solely on the aptamer sequence. In general, properly designed aptamers may exhibit high immunologic compatibility and are potential candidates for repeated administration in cancer immunotherapy (Tong et al., 2024; Ren et al., 2026).

#### 3.3.2 Off-target effects

Off-target effects are an important safety concern during the development of aptamer therapeutics despite their high molecular specificity. There can be off-target interactions due to partial sequence complementarity, unspecific electrostatic interactions, unintended binding to structurally related proteins, or altered expression of the target in normal tissues. Therapeutic efficacy can be compromised, and the risk for unwanted biological effects can be elevated if the aptamer is employed as a targeted delivery system for cytotoxic drugs or gene-editing systems, respectively. However, with the development of novel technologies such as Cell-SELEX, *in vivo* SELEX, HTS, and AI for sequence optimization, target selectivity has been significantly improved by removing non-specific binders and by designing sequences that more closely match the target (Li C. et al., 2025; Wilbanks et al., 2026). Furthermore, extensive pre-clinical assessment by multi-omics profiling, proteomic screening, and tissue cross-reactivity tests is becoming more common to discover and reduce off-target interactions before clinical translation (Wilbanks et al., 2025; Cheng et al., 2026). Although aptamers exhibit high molecular specificity, they still pose an important safety concern regarding off-target effects. The unintended effects can occur in multiple ways, none of which is mutually exclusive, such as partial sequence complementarity to endogenous nucleic acids, non-specific electrostatic interactions with plasma or cell-surface proteins, binding to structurally related proteins or receptors, and target expression in normal tissues (Liu et al., 2023; Jo et al., 2025). Additionally, aptamer folding can generate secondary or tertiary motifs that bind to unintended molecular surfaces. Chemical modification can change the charge and conformation of aptamers, and multimerization or aptamer-nanoparticle conjugation can alter protein binding, cellular uptake, and biodistribution. The effects can be especially significant when the aptamer is the delivery ligand, as even rare binding to normal tissues can lead to the accumulation and internalization of a cytotoxic, immunostimulatory, gene-silencing, or gene-editing payload (Benedetto et al., 2025; Shah et al., 2026). The unintended uptake can also lead to sequence-dependent modulation of transcripts for oligonucleotide cargos or to innate immune activation, while uptake by receptor-expressing normal cells can cause tissue injury in the event of premature extracellular release of cytotoxic payloads (Zhou C. et al., 2025). Therefore, off-target safety assessment should be based on the entire therapeutic construct–aptamer sequence and structure, linker stability, payload release, target distribution, and intracellular trafficking.

#### 3.3.3 Nuclease degradation

One key challenge to the use of unmodified aptamers, especially RNA aptamers, as therapeutics *in vivo* is their rapid degradation by nucleases. Exonucleases and endonucleases present in plasma can readily degrade phosphodiester bonds, thereby reducing plasma half-life and therapeutic efficacy (Dong X. et al., 2024; Kehrli et al., 2025). To overcome this drawback, many chemical engineering methods have been developed, including the use of 2′-F, 2′-OMe, and LNA nucleotides; a phosphorothioate backbone; terminal capping; circularization; and the development of spiegelmers (Liu Z. et al., 2025; Wen J. et al., 2025). Aptamers can also be encapsulated in nanoparticles or conjugated to PEG, lipids, or albumin-binding molecules to further shield them from enzymatic degradation and enhance pharmacokinetic properties. These improvements have greatly enhanced the *in vivo* stability of therapeutic aptamers and are crucial to their successful future clinical use (Yang S. et al., 2025; Ren et al., 2026).

#### 3.3.4 Clinical safety considerations

Based on current clinical data, aptamer therapeutics have a good safety profile, with most adverse events being mild or moderate and manageable. They are highly specific for their targets and have a low immunogenicity profile, which allows them to cause less systemic toxicity than many conventional chemotherapeutics and some biologics (Giordano et al., 2026; Khanani et al., 2026). But the clinical safety of the aptamer depends not only on the aptamer itself but also on its modifications, the attached payloads, and the delivery platforms. Another important safety issue with aptamer therapeutics is payload-associated toxicity, which arises from a mechanism distinct from that of the therapeutic agent. The toxicity profile of an aptamer–payload construct may not only include the toxicity of the target, but also include the intrinsic pharmacology of the payload, the chemistry of the coupling, the stability of the linker used, the intracellular trafficking of the aptamer, and the degree of premature release of the payload (Su et al., 2025). Cytotoxic drugs (e.g., topoisomerase inhibitors, tubulin inhibitors, antimetabolites) can be cytotoxic even if they are released before reaching the target cell, and siRNA, antisense, miRNA, or gene-editing cargos can induce unintended gene modulation or long-lasting pharmacodynamic effects after non-specific uptake by cells (Manabe et al., 2026). Another possibility for immunostimulatory cargo is the production of additional cytokines upon delivery to immune cells or endosomal compartments outside the intended site. Formulations using nanoparticles or lipids can cause additional toxicities due to the carrier type, complement activation, altered biodistribution, or accumulation in organs of clearance (Liang et al., 2026; Xie et al., 2026). Thus, characterization of the aptamer, the linker, the payload, the carrier, and the intact conjugate, such as free-payload exposure, tissue distribution (both target and non-target), intracellular release, organ-specific toxicity, immunotoxicity, and dose-dependent pharmacodynamic effects, may all be investigated separately (Su et al., 2025). In recent years, the integrated assessment of pharmacokinetic, toxicokinetic, biodistribution, and repeated-dose toxicity for an Sgc8c-monomethyl auristatin E–drug conjugate in a rodent-to-non-human primate translational study has highlighted the essential need for such studies to advance ApDCs toward clinical translation (Tao et al., 2026). Hence, detailed pre-clinical toxicology studies and well-designed clinical trials are still needed to assess biodistribution, immunotoxicity, pharmacokinetics, organ toxicity, and long-term toxicity. The therapeutic index is likely to be further enhanced through continued optimization of aptamer chemistry and delivery systems, thereby aiding the wider clinical use of aptamer-based cancer therapy.

## 4 Aptamer-mediated molecular targeting in cancer

Precise molecular recognition of disease-specific targets associated with tumor initiation, progression, metastasis, and immune evasion is the key to the therapeutic success of aptamer-based cancer therapy. Due to their high affinity, outstanding specificity, and ease of chemical engineering, aptamers can selectively bind to a wide range of cancer-associated molecules, not only on the surfaces of cancer cells but also in the TME (Quintavalle et al., 2025; Su et al., 2025). The targeting capability allows aptamers to be directly used in therapy, targeted drug delivery platforms, molecular imaging probes, and immunomodulatory platforms, without inducing significant off-target toxicities. Recent developments in aptamer engineering have broadened their range of applications beyond targeting cancer cells to include cancer stem cells (CSCs), tumor vasculature, and stromal elements, all of which play key roles in therapeutic resistance and disease recurrence (Liu et al., 2025c; Liu et al., 2025 J.). Here, the major molecular targets that therapeutic aptamers can engage are discussed, with a focus on their utility in precision oncology and cancer immunotherapy through selective targeting of tumor cell-surface biomarkers, tumor stem cells, tumor vasculature, and stromal cells.

### 4.1 Tumor cell surface biomarkers

The most common tumor cell surface biomarker targets are those that are highly expressed in cancer cells, but not in normal cells. They may be targeted for highly selective therapeutic intervention via an aptamer. Such membrane-associated proteins have been implicated in many aspects of oncogenic activity, including cell proliferation, survival, invasion, metastasis, angiogenesis, and immune evasion. They are considered promising targets in precision oncology. Probes targeting biomarker molecules can be used as receptor antagonists or agonists, and for targeted delivery of chemotherapeutic agents, nucleic acids, proteins, nanoparticles, and imaging probes. The aptamers are more amenable to chemical modification, more tissue-penetrating, and less immunogenic than monoclonal antibodies, which is useful for efficient localization and transport of cargo into the cellular interior of tumors (Su et al., 2025; Xu F. et al., 2025). Highly specific aptamers to clinically relevant biomarkers such as EpCAM, HER2, EGFR, PSMA, MUC1, nucleolin, and CD44 are being developed and increasingly tested in pre-clinical studies and early-phase clinical translation for targeted cancer therapy and immuno-oncology (Table 4) (Caliendo et al., 2025; Liu J. et al., 2025).

**TABLE 4:** Major tumor cell surface biomarkers targeted by therapeutic aptamers in precision cancer therapy.

Biomarker	Physiological function	Cancer association	Representative cancer types	Therapeutic relevance of aptamers	References
EpCAM	Cell adhesion, epithelial integrity, proliferation	Frequently overexpressed in epithelial cancers; associated with tumor initiation, metastasis, and circulating tumor cells	Colorectal, breast, prostate, gastric, pancreatic, ovarian	Targeted drug delivery, circulating tumor cell capture, molecular imaging, aptamer-drug conjugates	(Sarkar et al., 2024; Huang et al., 2025b)
HER2	Receptor tyrosine kinase regulating cell growth and survival	HER2 amplification drives aggressive tumor growth and therapeutic resistance	Breast, gastric, gastroesophageal, ovarian	Receptor inhibition, targeted chemotherapy delivery, nanoparticle targeting, imaging	(Katsuba et al., 2025; Kavruk et al., 2025; Pawar et al., 2025)
EGFR	Controls proliferation, differentiation, migration, and survival	Aberrant activation promotes uncontrolled proliferation and metastasis	Non-small cell lung cancer, glioblastoma, colorectal, head and neck, triple-negative breast cancer	Inhibition of EGFR signaling, selective drug delivery, siRNA transport, radio-sensitization	(Thomas et al., 2024a; Li et al., 2025h)
PSMA	Membrane glutamate carboxypeptidase involved in folate metabolism	Highly expressed in advanced prostate cancer and tumor neovasculature	Prostate cancer; neovasculature of several solid tumors	Precision drug delivery, radionuclide therapy, molecular imaging, theranostics	(Noble et al., 2024; Dhull et al., 2025)
MUC1	Transmembrane glycoprotein involved in epithelial protection and signaling	Aberrantly glycosylated and overexpressed, promoting invasion and immune evasion	Breast, pancreatic, ovarian, lung, colorectal	Targeted delivery of drugs and nucleic acids, immunotherapy, cancer imaging	(Jamshidi et al., 2024; Gharehbaba et al., 2025)
Nucleolin	Ribosome biogenesis, RNA metabolism, chromatin regulation; translocated to cancer cell surface	Cell-surface overexpression correlates with rapid proliferation, angiogenesis, and metastasis	Breast, lung, pancreatic, glioma, leukemia	Internalizing aptamer platform for intracellular drug delivery, imaging, targeted nanomedicine	(Ravi Mythili et al., 2025; Wang et al., 2025b)
CD44	Hyaluronic acid receptor regulating adhesion, migration, and stem cell maintenance	Marker of CSCs; associated with metastasis, recurrence, and drug resistance	Breast, colorectal, gastric, pancreatic, ovarian, head and neck	Targeting CSCs, inhibition of metastasis, selective drug delivery, combination immunotherapy	(Caliendo et al., 2025; Yang et al., 2025b)

Clinically relevant surface tumor cell biomarkers that have been targeted for aptamer-based precision cancer therapy. These biomarkers are associated with key oncogenic mechanisms, including proliferation, metastasis, angiogenesis, stemness, and immune evasion. Aptamers that bind these molecules have shown promise as therapeutic agents, targeted delivery ligands, molecular imaging probes, and theranostic platforms, and are increasingly being used in precision oncology and cancer immunotherapy.

### 4.2 Targeting CSCs

CSCs are a small population of tumor cells that can self-renew, differentiate into multiple lineages, and initiate tumors. The cells play pivotal roles in tumor initiation, dissemination, therapeutic resistance, immune evasion, and disease recurrence, and are therefore important therapeutic targets in precision cancer therapy. CSCs exhibit enhanced expression of certain surface markers (CD44, CD133, EpCAM, CD24, CD90, and aldehyde dehydrogenase) that distinguish them from differentiated tumor cells and enable selective targeting (Figure 2) (Wei J.-R. et al., 2024; Xu et al., 2024). The characteristics of high binding specificity, excellent tissue penetration, low immunogenicity, and ease of chemical modification make aptamers very promising ligands for CSC-directed therapy. Aptamers can specifically target certain biomarkers associated with CSC, inhibit signaling pathways associated with stemness, and block the tumor-initiating process. Aptamers can also be used as targeted vehicles for chemotherapeutics, siRNAs, miRNAs, CRISPR/Cas systems, and nanomedicines, thereby enhancing therapeutic selectivity and reducing systemic toxicity (Caliendo et al., 2025; Li W. et al., 2025).

**FIGURE 2:**
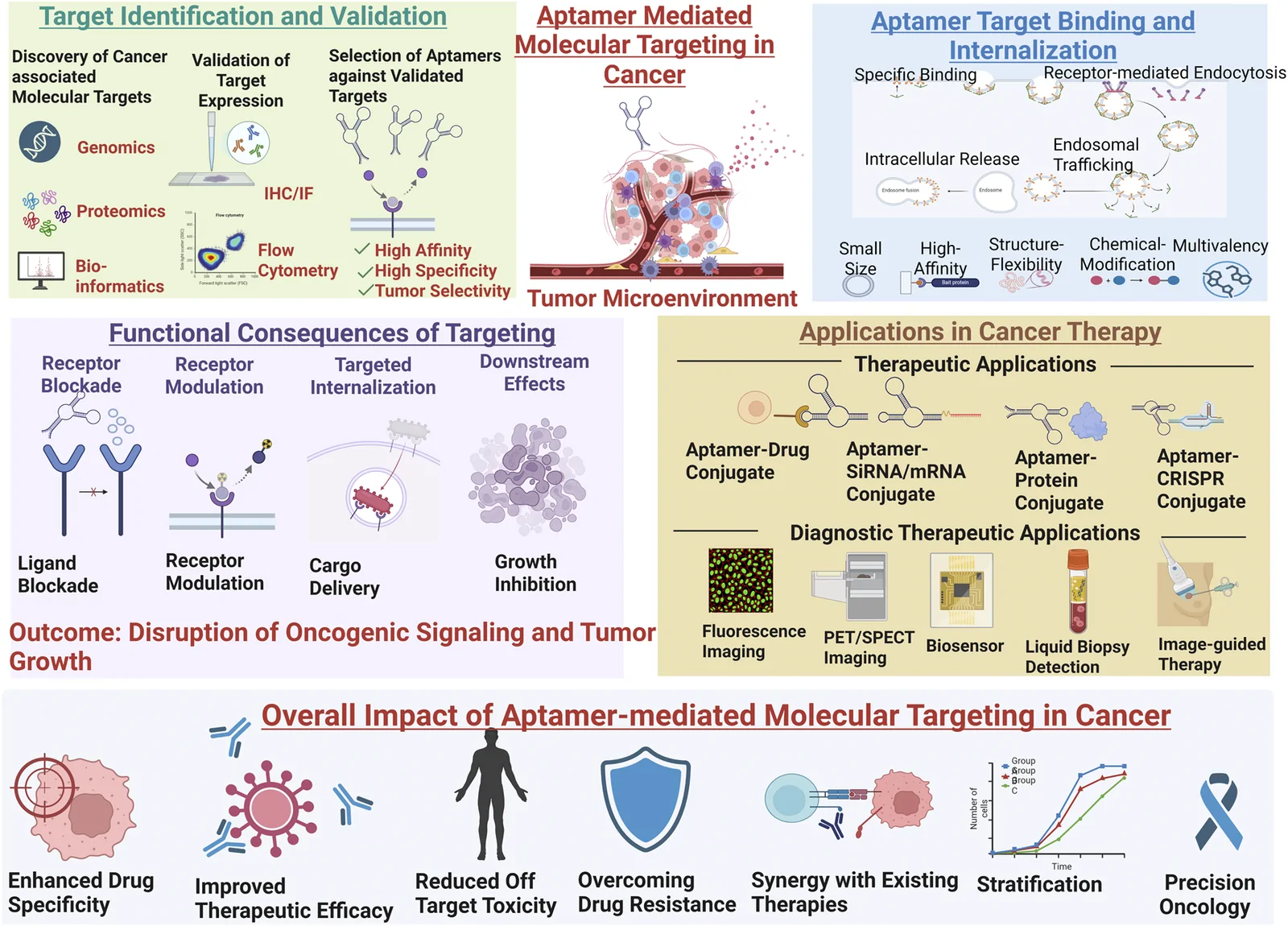
Aptamer-mediated molecular targeting strategies in cancer: selective recognition of tumor cells and the TME.

Recent developments have also broadened the scope of aptamer platforms from recognition of CSCs to multifunctional therapeutic systems that cannot only kill CSCs but also remodel the TME (Liu et al., 2025c; Zuo et al., 2025). Aptamer-mediated delivery systems have shown increased intracellular delivery of therapeutic payloads in CSCs, resulting in inhibition of several key pathways associated with stemness regulation, including Wnt/β-catenin, Notch, Hedgehog, PI3K/AKT/mTOR, and STAT3, which are commonly dysregulated in treatment-resistant tumors (Wei Y. et al., 2024; Dwivedy et al., 2025; Li et al., 2025g). Additionally, bispecific and multivalent aptamers are being engineered to enhance targeting efficiency, intratumoral heterogeneity, and drug aggregation within CSC niches, while aptamer-functionalized exosomes are under development to achieve more efficient drug delivery to these niches. Additionally, evidence has emerged that targeting CSCs with aptamers can modulate the immune system to enhance the effects of ICI and standard chemotherapy by dampening CSC-mediated immunosuppression and blocking tumor recurrence (Li X. et al., 2025; Liu et al., 2025c; Wang et al., 2026a). Thus, targeting CSCs with aptamers is a potentially effective approach to obtain long-lasting therapeutic effects and lower the risk of recurrence in precision cancer immunotherapy (Figure 2).

### 4.3 Targeting tumor vasculature

The tumor vasculature is an attractive therapeutic target because it provides oxygen, nutrients, and signaling molecules to rapidly proliferating tumor cells, thereby enabling tumor growth, metastasis, and immune escape. In contrast to normal tissues, tumor blood vessels are structurally abnormal, highly permeable, and abundant in angiogenesis that is mainly stimulated by vascular endothelial growth factor (VEGF), vascular endothelial growth factor receptor-2 (VEGFR2), integrins (especially αvβ3), platelet-derived growth factor (PDGF), and endoglin (CD105) (Takada et al., 2024; Zhu J. et al., 2024; Bastola et al., 2025). Targeting these angiogenic mediators with aptamers may inhibit endothelial cell proliferation, migration, and neovascularization, thereby limiting tumor perfusion and metastatic dissemination (Figure 2). The small size and high specificity of aptamers enable them to penetrate abnormal tumor vessels and specifically recognize angiogenic endothelial markers without causing significant effects in normal vasculature, an advantage over conventional anti-angiogenic biologics (Mi et al., 2005; Teng et al., 2023).

In addition to directly inhibiting angiogenesis, aptamers targeting vessels have proven to be highly effective ligands for vascular-targeted drug delivery and vascular normalization. Anti-VEGF, anti-VEGFR2, anti-nucleolin, anti-integrin αvβ3, and anti-endoglin aptamers have been engineered into nanoparticles, liposomes, exosomes, and other multifunctional delivery vehicles for the selective targeting of tumor-associated endothelial cells and the delivery of chemotherapeutic agents, siRNA, miRNA, and immunomodulatory cargo (Feng Z. et al., 2025; Kim S. et al., 2025; Chen et al., 2026b). This selective mechanism allows for higher drug levels in the local area while reducing toxicity elsewhere in the body. In addition, normalization of the dysfunctional tumor vasculature by anti-angiogenic aptamers results in improved oxygenation and increased infiltration potential of cytotoxic T cells. It enables the delivery of immune checkpoint inhibitors (ICIs) and other anti-cancer drugs (Di Tacchio et al., 2019; Feng Z. et al., 2025). Thus, aptamer targeting of tumor vessels is a promising approach for simultaneously blocking angiogenesis, facilitating the delivery of therapeutic agents to the tumor, and enhancing cancer immunotherapy (Figure 2).

### 4.4 Stromal cell targeting

The TME is a complex, ever-changing part of the tumor that is crucial for cancer progression, therapy resistance, and immunosuppression. It is composed of a network of cells and cell components, including cancer-associated fibroblasts (CAFs), tumor-associated macrophages (TAMs), mesenchymal stromal cells, pericytes, endothelial cells, and ECM, that interact in numerous ways to create a pro-tumorigenic microenvironment (Lu et al., 2025; Peura et al., 2025). Growth factors, inflammatory cytokines, chemokines, matrix-remodeling enzymes, and immunosuppressive mediators, such as TGF-β, IL-6, CXCL12, and matrix metalloproteinases, secreted by stromal cells, stimulate tumor proliferation, invasion, metastasis, angiogenesis, and immune evasion (Ghazimoradi and Babashah, 2025; Mitsui et al., 2025). Aptamers targeted to stromal targets such as fibroblast activation protein, CXCL12, PDGFR, TGF-β, and immune checkpoint molecules offer a highly precise approach to disrupting tumor-supportive interactions while leaving normal stromal tissue largely unscathed (Zhang X. et al., 2023; Liu et al., 2025c).

In recent years, stromal-targeting aptamers have been developed into multifunctional therapeutic platforms that remodel the TME. Aptamer-functionalized nanocarriers and aptamer–cargo conjugates have been used to selectively deliver chemotherapeutics, siRNAs, miRNAs, and immunostimulatory agents to CAFs, TAMs, and other stromal populations, resulting in decreased ECM deposition, removal of physical barriers to drug penetration, and reversal of local immunosuppression (Ibba et al., 2023; Quintavalle et al., 2025). Furthermore, stromal-targeting aptamers can boost the antitumor immune response by reprogramming macrophages to a pro-inflammatory M1 phenotype, blocking CAF-induced fibrosis, and interfering with the CXCL12–CXCR4 axis, thereby preventing the infiltration of cytotoxic T cells at the tumor site (Qian et al., 2022; Yang Q. et al., 2024). The various functions of these cells enhance immune cell infiltration and complement other immunotherapies, such as immune checkpoint blockade (ICB), making stromal cell targeting an increasingly attractive component of next-generation aptamer-based precision cancer immunotherapy (Liu et al., 2025c).

Aptamers are obtained against clinically relevant tumor-associated biomarkers identified through genomic, proteomic, and molecular profiling, enabling high-affinity, high-specificity recognition of cancer cell-surface targets, including EGFR, HER2, EpCAM, PSMA, CD44, MUC1, and nucleolin (Katsuba et al., 2025; Liu K. et al., 2025). In addition to differentiated tumor cells, aptamers can be designed to specifically target CSCs based on CSC-associated molecular markers, such as CD44, CD133, EpCAM, and aldehyde dehydrogenase, thereby blocking tumor initiation, therapeutic resistance, and disease recurrence (Grover et al., 2023; Caliendo et al., 2025). Target recognition is followed by aptamer binding to the cell-surface receptor, leading to receptor-mediated endocytosis, enabling intracellular trafficking and selective delivery of the therapeutic cargo with high tumor specificity and low off-target accumulation (Benedetto et al., 2025; Yang K. et al., 2025). The binding of aptamers leads to several pharmacological effects, such as receptor antagonism or agonism, inhibition of the interaction between the receptor and the ligand, regulation of intracellular signaling pathways, and, importantly, targeted delivery of drugs, nucleic acids, proteins, peptides, or genome editing systems to the interior of cells, ultimately inhibiting tumor growth and survival (Ning et al., 2025; Vasconcelos et al., 2025). In addition to recognizing malignant cells, aptamers also target components of the tumor vasculature, notably angiogenic molecules such as VEGFR2, VEGFR2-VEGF dimers, endoglin (CD105), and integrin αvβ3, thereby inhibiting angiogenesis and enhancing delivery of therapeutic agents to the tumor (Feng Z. et al., 2025; Maciulis et al., 2025). Moreover, aptamers targeting stromal components such as CAFs, TAMs, fibroblast activation protein, CXCL12, PDGFR, and TGF-β signaling remodel the TME, promote immune cell infiltration, and overcome stromal-mediated therapeutic resistance (Giordano et al., 2024; Liu et al., 2025c). Molecular targeting strategies have found wide applications in therapeutic and diagnostic fields, such as ApDCs, aptamer-mediated delivery of RNA and CRISPR, protein and peptide targeting, molecular imaging, biosensing, liquid biopsy, and theranostics. If used together, the targeting specificity of aptamers to tumors, the greater effect of the targeted compounds, reduced systemic side effects, reduced drug resistance, and precision oncology through simultaneous targeting of cancer cells and the TME create a significant advantage (Su et al., 2025).

## 5 Aptamers as precision drug delivery systems

The application of aptamers as targeted drug-delivery vehicles has broadened their therapeutic capabilities beyond molecular recognition, leading to their use as precision cancer therapeutics. Aptamers possess low immunogenicity, are highly specific to their targets, and can be chemically modified. They can specifically deliver a wide array of therapeutic payloads to tumor cells and components of the TME without significant off-target toxicity or systemic adverse effects (Laowichuwakonnukul et al., 2025; Wu Y. et al., 2025). Compared with passive drug delivery systems, aptamer-based systems also leverage receptor-mediated targeting and cellular uptake to deliver therapeutic agents into cells, enhance pharmacokinetic properties, and bypass biological barriers encountered by conventional anticancer strategies (Su et al., 2025; Xu F. et al., 2025). Recent progress in nucleic acid engineering, nanotechnology, and biomaterials has enabled the design of multifunctional aptamer-based platforms, including aptamer–drug, aptamer–siRNA, and aptamer–miRNA delivery systems, as well as aptamer–protein, peptide, and aptamer-guided CRISPR/Cas delivery systems (Elsharkasy et al., 2025; Wu Y. et al., 2025). All of these precision delivery approaches have the potential to increase therapeutic efficacy, reduce systemic toxicity, and create greater opportunities for personalized cancer immunotherapies, making aptamers a promising next-generation targeted delivery vehicle in precision oncology.

### 5.1 ApDCs

The high molecular specificity of aptamers, coupled with the cytotoxic activity of anticancer drugs, is making ApDCs a promising class of targeted therapeutics for drug delivery to tumor cells while minimizing systemic drug toxicity (Su et al., 2025). ApDCs are similar in function to antibody–drug conjugates and use aptamers as the targeting ligand that binds tumor-associated biomarkers, including EpCAM, HER2, EGFR, PSMA, MUC1, CD44, and nucleolin, thereby triggering receptor-mediated internalization and release of the therapeutic agent into the cytoplasm (Tao et al., 2026; Woo et al., 2026). Various chemotherapeutic agents have been linked with cleavable or non-cleavable linkers designed to be activated by tumor-specific stimuli such as pH changes, high levels of glutathione, reactive oxygen species, or lysosomal enzymes. This focused approach may increase drug concentration at the tumor site, minimize exposure to non-cancerous tissue, and broaden the therapeutic window compared with standard systemic chemotherapy (Hu et al., 2025; Wu Y. et al., 2025).

In recent years, the design of ApDCs has targeted three main goals: increasing payload capacity, enhancing intracellular trafficking, and combining multifunctional therapeutic modalities. Nanotechnology-based ApDCs, such as liposomal, polymeric, DNA nanostructure, and exosome-based ApDCs, offer enhanced drug-loading capacity, extended circulation time, and targeted drug delivery, in addition to their aptamer-targeting ability (Makkar et al., 2025; Uner et al., 2025). Meanwhile, bispecific and multivalent aptamers targeting multiple tumor-associated receptors have been developed to achieve greater tumor selectivity and overcome receptor heterogeneity and acquired resistance. Moreover, ApDCs are being used in combination with other therapeutic modalities, such as ICIs, gene-silencing agents, and photodynamic and photothermal therapeutics, to achieve synergistic antitumor activity (Figure 3). While these hurdles still exist, the continued development of chemical conjugation technologies, biomaterials, and precision drug delivery systems ensures that the ApDC platform is poised to be a significant next-generation platform for targeted cancer treatment and immuno-oncology (Xu H. et al., 2025; Chi et al., 2026).

**FIGURE 3:**
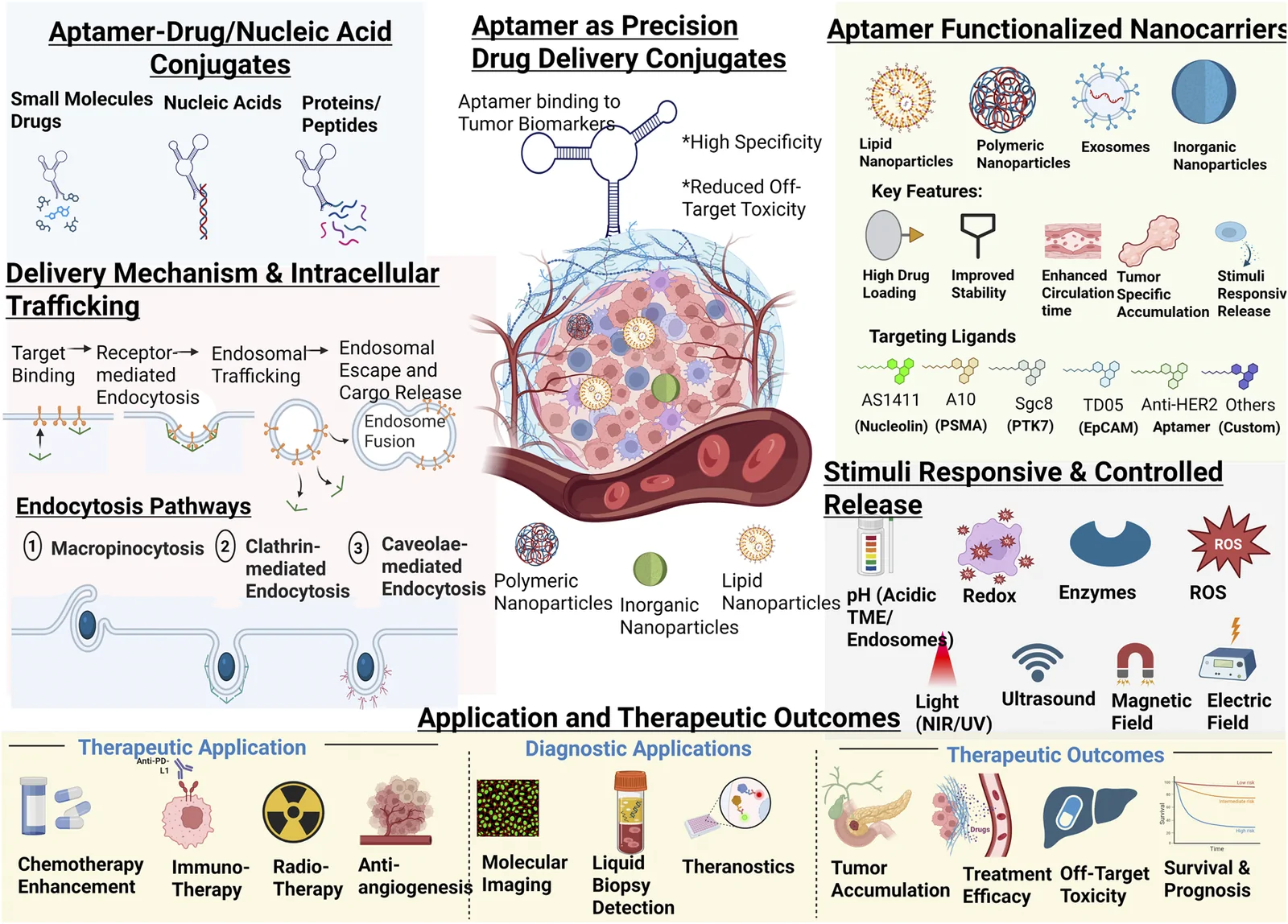
Aptamer-based precision drug delivery systems for targeted cancer therapy and immunotherapy.

### 5.2 Aptamer–siRNA conjugates

Aptamer–siRNA conjugates are a targeted gene-silencing platform that combines the cell-specific recognition of aptamers with the sequence-specific activity of RNA interference (RNAi) (Vasconcelos et al., 2025; Chen X. et al., 2026). In these chimeras, the aptamer binds a cell-surface receptor and promotes receptor-mediated endocytosis, after which the conjugate enters early and subsequently late endosomal compartments. However, cellular uptake does not necessarily translate into productive RNAi because a substantial fraction of internalized aptamer–siRNA molecules can remain trapped within endosomes and ultimately undergo lysosomal degradation (Tanaka et al., 2021; Zhang N. et al., 2022). Effective silencing requires escape from the endosomal compartment before lysosomal degradation, followed by appropriate processing of the siRNA and loading of the guide strand into the RNA-induced silencing complex (RISC) (Malong et al., 2025). Endosomal escape is therefore a major intracellular bottleneck in aptamer–siRNA delivery. Several approaches have been explored to enhance cytosolic release, including pH-responsive or ionizable lipids, fusogenic peptides, polycationic or protonatable polymers, polyhistidine-containing systems, and stimuli-responsive nanocarriers (Lee et al., 2024; Zhao L. et al., 2025). These systems can promote membrane destabilization through protonation, osmotic effects, membrane fusion, or transient disruption of the endosomal membrane; however, excessive membrane disruption may itself cause cellular toxicity. Importantly, the efficiency and mechanism of endosomal escape remain highly dependent on the architecture of the aptamer–siRNA construct and its delivery carrier, and efficient receptor-mediated internalization alone may not be interpreted as evidence of efficient cytosolic delivery (Table 5) (Tanno et al., 2023). A second major limitation is the *in vivo* instability of the complete aptamer–siRNA chimera. Both the aptamer and siRNA domains can undergo nuclease-mediated degradation in biological fluids, and cleavage of either domain can compromise receptor recognition, intracellular trafficking, or RNAi activity (Ni et al., 2015; Rosch et al., 2022). In addition, after cellular uptake, exposure to endosomal and lysosomal nucleases can degrade conjugates that fail to escape efficiently. Chemical stabilization strategies, including 2′-F, 2′-OMe, LNA, selected phosphorothioate linkages, terminal stabilization, and PEG or albumin-binding strategies, can improve resistance to extracellular degradation and prolong systemic exposure (Datta et al., 2024). However, the density and position of modifications must be optimized, as excessive modification can alter aptamer folding, siRNA processing, target recognition, or RISC activity. Importantly, improved serum stability does not necessarily indicate successful intracellular delivery. For example, albumin-binding aptamer–siRNA chimeras have demonstrated improved *in vivo* bioavailability while retaining gene-silencing activity, illustrating the importance of simultaneously optimizing systemic stability and functional intracellular delivery (Table 5) (Hoogenboezem et al., 2024). Thus, development of aptamer–siRNA therapeutics requires assessment of intact chimera stability, degradation products, tissue distribution, intracellular trafficking, cytosolic release, and functional target knockdown rather than relying solely on serum half-life measurements (Toshima et al., 2023; Ly et al., 2025).

**TABLE 5:** Aptamer-based precision drug delivery platforms in cancer therapy and immunotherapy.

Delivery platform	Therapeutic cargo	Primary therapeutic objective	Key advantages	Representative applications	References
ApDCs	Small-molecule chemotherapeutics and cytotoxic agents	Selective destruction of tumor cells through receptor-mediated drug delivery	High tumor selectivity, reduced systemic toxicity, controlled intracellular drug release	Precision chemotherapy, combination immunotherapy, theranostics	(Su et al., 2025; Wu et al., 2025a)
Aptamer–siRNA Conjugates	siRNA	Sequence-specific silencing of oncogenes, resistance-associated genes, and immune regulators	Precise gene knockdown, minimal off-target exposure, improved intracellular RNA delivery	Gene therapy, reversal of drug resistance, immune checkpoint modulation	(Rojo et al., 2025; Zeng et al., 2025)
Aptamer–miRNA Delivery Systems	miRNA mimics and anti-miRNA oligonucleotides (antagomiRs)	Restoration of dysregulated post-transcriptional gene regulation	Simultaneous regulation of multiple signaling pathways, enhanced therapeutic specificity	Suppression of metastasis, cancer stem cell inhibition, immune microenvironment remodeling	(Fang et al., 2025b; Laowichuwakonnukul et al., 2025)
Aptamer–Protein and Peptide Conjugates	Cytokines, enzymes, apoptotic proteins, immune-modulating peptides	Targeted delivery of biologically active macromolecules to tumors	Preservation of protein activity, localized immune modulation, reduced systemic adverse effects	Cytokine-based immunotherapy, apoptosis induction, targeted protein therapeutics	(Demirbolat et al., 2026; Zhang et al., 2026b)
Aptamer–CRISPR Delivery Systems	CRISPR/Cas nucleases, guide RNAs, ribonucleoproteins (RNPs), base editors, prime editors	Targeted genome and epigenome engineering	Cell-specific genome editing, reduced off-target delivery, compatibility with non-viral vectors	Precision gene editing, immuno-oncology, correction of oncogenic and resistance-associated pathways	(Sarkar et al., 2024; Liao et al., 2026)

Despite receptor-directed targeting, off-target gene silencing remains an important safety concern because the siRNA component can interact with unintended transcripts once it enters the RNAi machinery. In addition to imperfect sequence complementarity to unintended mRNAs, the guide-strand seed region, particularly nucleotides 2–8, can mediate miRNA-like repression of transcripts containing complementary sequences, especially within 3′ untranslated regions (Wang and Bartel, 2024). Off-target effects may also arise from passenger-strand activity, excessive intracellular siRNA concentrations, or sequence-dependent interactions with the RNAi machinery. Aptamer targeting can reduce nonspecific delivery to non-target cells but does not eliminate sequence-dependent off-target silencing in cells that successfully internalize the conjugate (Sahoo et al., 2024; Agarwal et al., 2025). This distinction is particularly important when the targeted receptor is expressed at low levels in normal tissues or when the therapeutic siRNA regulates genes involved in cell survival, immune signaling, or other essential physiological pathways. Therefore, siRNA sequence selection may incorporate genome-wide off-target prediction and transcriptomic analysis, while experimental validation should include multiple independent siRNA sequences, appropriate scrambled controls, dose–response analysis, rescue experiments where feasible, and assessment of global transcriptomic changes (Subramanian et al., 2023; Long et al., 2024). Chemical modification of the guide-strand seed region can further reduce seed-mediated off-target effects in appropriately designed constructs. Consequently, the therapeutic specificity of aptamer–siRNA conjugates may be evaluated at both levels—aptamer-mediated cellular targeting and siRNA-mediated sequence specificity—rather than assuming that receptor targeting alone guarantees molecular specificity (Vasconcelos et al., 2025).

Aptamer–siRNA conjugates have been investigated against clinically relevant targets, including PSMA, EpCAM, nucleolin, HER2, EGFR, and MUC1, with siRNAs directed against genes such as PLK1, BCL2, survivin (BIRC5), STAT3, VEGF, KRAS, and PD-L1 showing antitumor activity in preclinical models (Hao et al., 2024; Salve et al., 2024). Recent studies continue to demonstrate the feasibility of serum-stable aptamer–siRNA constructs for systemic administration, including PSMA-targeted chimeras targeting androgen receptor variants in resistant prostate cancer models (Xue et al., 2024). Nevertheless, successful clinical translation may require simultaneous optimization of receptor-mediated uptake, endosomal escape, nuclease resistance, intracellular processing, and sequence-specific silencing while rigorously excluding unintended gene regulation and tissue exposure.

### 5.3 Aptamer–miRNA delivery

Given the cell-specific targeting ability of aptamers and the gene-regulatory activity of miRNAs, the aptamer-mediated delivery of miRNAs is a promising approach for precision cancer therapy. miRNAs are small, non-coding RNAs of about 20–24 nucleotides in length, which control the expression of a gene at the post-transcriptional level by promoting the degradation of the mRNA or inhibiting translation (Esposito et al., 2014; Fang et al., 2025b). Numerous cancers are associated with dysregulated miRNA expression, which is linked to uncontrolled cell growth, evasion of apoptosis, metastasis, angiogenesis, immune evasion, and resistance to therapy. Selectively targeting tumor cells with aptamers specific for tumor-associated receptors like EpCAM, PSMA, HER2, EGFR, nucleolin, and MUC1, the therapeutic molecules, namely miRNA mimics or anti-miRNA oligonucleotides (antagomiRs), are delivered to the malignant cells by endocytosis, and the tumor suppressive miRNAs are restored or oncogenic miRNAs are inhibited, respectively (Table 5) (Esposito et al., 2014; Perepelyuk et al., 2016). Such a targeted approach increases cellular uptake, enhances therapeutic specificity, and reduces systemic uptake compared with conventional miRNA delivery approaches (Figure 3).

In recent years, the engineering of aptamers to miRNAs has centered on enhancing their stability, intracellular targeting, and therapeutic activity by incorporating chemical modifications and nanotechnology-based delivery systems (Fang et al., 2025b). To promote nuclease resistance and increase blood circulation, a variety of strategies have been used to stabilize aptamer–miRNA chimeras, including 2′-F, 2′-OMe, LNA, phosphorothioate, and PEGylation (An et al., 2026). In addition, lipid nanoparticles, polymeric nanoparticles, DNA nanostructures, extracellular vesicles, and stimuli-responsive nanocarriers have been used to enhance endosomal escape and the release of cargo miRNA into the cytoplasm (Abdelaal et al., 2024). In terms of therapeutic applications, the delivery of tumor-suppressive miRNAs, including miR-34a, let-7, miR-143, miR-145, and miR-200 family members by aptamers has been shown to exert strong antitumor effects in pre-clinical models via inhibition of oncogenic pathways, restoration of the E-MT, inhibition of cancer stemness, and sensitization to chemotherapy and ICB (Esposito et al., 2014; Wang et al., 2015; Xiong et al., 2025). In contrast, the inhibition of oncogenic miRNAs such as miR-21, miR-155, and miR-221 has shown strong anti-tumor effects in these models. In the context of precision oncology and next-generation cancer immunotherapy, these developments position the aptamer–miRNA delivery system favorably and make it likely to become an increasingly significant platform (Liao et al., 2023; Fang et al., 2025b).

### 5.4 Aptamer–protein and peptide conjugates

The aptamer–protein and aptamer–peptide conjugates are a broad class of targeted therapeutics that combine the high target specificity of aptamers with the biology of proteins and peptides. In such constructs, aptamers are precision-targeted ligands that specifically bind tumor-associated receptors, enabling the delivery of therapeutic proteins, cytokines, enzymes, antibodies, or bioactive peptides to malignant tissues without systemic toxicity (Wang Y. et al., 2024; Li L. et al., 2025). This targeted approach may increase therapeutic efficacy by raising drug concentration in the target area, enhancing receptor-mediated internalization, and reducing off-target exposure. Aptamers targeting clinically relevant targets like EGFR, HER2, EpCAM, nucleolin, PSMA, and PD-L1 have been used to deliver a variety of protein and peptide cargos, such as tumor necrosis factor-related apoptosis-inducing ligand (TRAIL), granzyme B, ribosome-inactivating proteins (e.g., gelonin and saporin), cytokines (e.g., IL-2 and IL-12), and immune-modulating peptides (Chu et al., 2006; Wang Y. et al., 2024; Demirbolat et al., 2026). These conjugates leverage receptor-specific uptake mechanisms to increase both the uptake and the activity of the protein/peptide payload within the cell and/or at the tumor site (Table 5).

In the past few years, aptamer–protein and peptide conjugates have evolved into a multifunctional immunotherapeutic platform capable of simultaneously targeting tumors and modulating the immune microenvironment. Aptamers have been engineered to selectively home to the tumor, thereby decreasing the systemic toxicity of the cytokines, and to activate tumor-reactive CD8^+^ T cells, NK cells, and dendritic cells (DCs) (Tan et al., 2025; Wen N. et al., 2025). Likewise, tumor cell-specific delivery of pro-apoptotic peptides and immune-modulating proteins via aptamers has been shown to increase tumor cell death, block metastasis, and enhance ICB in pre-clinical studies (Zheng et al., 2022; Marzano et al., 2026). Concurrently, the design of bispecific aptamer–protein conjugates, the development of site-specific conjugation chemistries, and the development of nanotechnology-based technologies have led to improved conjugate stability, controlled payload delivery, and pharmacokinetic properties. In conclusion, the innovations presented here showcase aptamer–protein and peptide conjugates as a promising approach for precision cancer therapy, providing a platform with targeted drug-delivery capability, immunomodulatory effects, and combination therapeutic efficacy (Wu Y. et al., 2025; Zuo et al., 2025).

### 5.5 Aptamer–CRISPR delivery systems

Aptamer–CRISPR delivery systems are a new frontier in precision cancer therapeutics, combining the cell-specific targeting of aptamers with the genome–and transcriptome-editing capabilities of CRISPR technologies (Liao et al., 2026). One of the key challenges of CRISPR-based therapy is the efficient and precise delivery of CRISPR components to target cells with minimal off-target editing to healthy tissues. Aptamers alleviate this drawback by acting as molecular targeting ligands that bind to receptors found on tumor cells and facilitate receptor-mediated internalization of CRISPR cargoes (Sarkar et al., 2024). Aptamers have been used to transport CRISPR/Cas9, CRISPR/Cas12a, CRISPR interference (CRISPRi), CRISPR activation (CRISPRa), and recently, base editing and prime editing systems as plasmid DNA, mRNA, ribonucleoprotein complexes, or guide RNAs, depending on the therapeutic goal (Collantes et al., 2021; Geilenkeuser et al., 2025). These platforms enable targeted editing of oncogenes, tumor suppressor pathways, immune checkpoint molecules, and therapeutic resistance genes to increase treatment specificity and minimize systemic toxicity by selecting appropriate biomarkers, including EpCAM, HER2, EGFR, PSMA, nucleolin, and CD44 (Liao et al., 2026).

Therapeutic aptamers bind highly selectively to surface-specific tumor markers such as EpCAM, HER2, EGFR, PSMA, MUC1, nucleolin, and CD44, and mediate their internalization to selectively deliver a variety of therapeutic payloads with low off-target toxicity (Liu et al., 2025d). The controlled intracellular drug release by ApDCs and their ability to selectively target cytotoxic agents to malignant cells have the potential to improve therapeutic efficacy (Figure 3). Aptamer-siRNA conjugates enable delivery of siRNAs to specific receptors and subsequent RNAi-mediated silencing of oncogenes, immune checkpoint molecules, and therapy resistance-associated genes (Hao et al., 2024; Su et al., 2025). Aptamer-mediated miRNA delivery restores dysregulated post-transcriptional gene regulation by selectively delivering tumor-suppressive miRNA mimics or inhibiting the expression of oncogenic miRNAs, thereby suppressing tumor development and metastatic signaling. Aptamer–protein and peptide conjugates can be used for targeted delivery of cytokines, enzymes, apoptotic proteins, and immune-modulating peptides, thereby increasing their biological activity at the target site and minimizing systemic toxicity (Demirbolat et al., 2026). Aptamer-guided CRISPR delivery systems deliver genome-editing components, such as Cas nucleases, guide RNAs, ribonucleoprotein complexes, base editors, and prime editors, to precisely edit cancer- and immune-related genes (Table 5). These platforms can be used together to enhance tumor selectivity, intracellular delivery, pharmacokinetic properties, and therapeutic effect, and can be combined with chemotherapy, immunotherapy, gene therapy, and nanomedicine for precision oncology (Geilenkeuser et al., 2025; Yıldırım, 2025).

Recent studies have focused on the design of multifunctional aptamer-based nanocarriers that can overcome the biological obstacles inherent to CRISPR delivery, such as serum instability, inefficient trafficking to the cell interior, endosomal capture, and restricted nuclear access (Elsharkasy et al., 2025; Xu et al., 2026). Aptamers have been fused with lipid nanoparticles, polymeric nanoparticles, DNA nanostructures, extracellular vesicles, and stimuli-responsive biomaterials to improve the tumor-specific targeting, intracellular delivery, and genome-editing efficiency. In addition to directly disrupting oncogenic signaling pathways, targeted gene editing of molecules that regulate tumor immunity, such as PD-1, PD-L1, CTLA-4, TGF-β, and CXCR4, is also being intensively investigated to boost anti-tumor immunity and reverse immune therapy resistance in the context of aptamer-mediated CRISPR delivery (He et al., 2020; Elsharkasy et al., 2025; Liao et al., 2026). The future of the aptamer-CRISPR platform is incredibly promising for highly personalized cancer therapeutics and precision immuno-oncology, though obstacles remain, including challenges in large-scale manufacturing, editing efficiency, long-term safety, and regulatory considerations (Table 5).

The aptamers' ability to recognize and selectively deliver a variety of therapeutic agents, including cytotoxic drugs, regulatory RNAs, proteins, peptides, and gene-editing agents, to tumor cells or the TME (Su et al., 2025). They function in complementary ways that can facilitate the development of individual treatment plans and offer potential for synergistic combination with other targeted therapeutic strategies, such as immunotherapy and nanomedicine.

## 6 Engineering aptamers for cancer immunotherapy

The versatility of aptamers has underscored their potential as a platform for next-generation cancer immunotherapy, both for modulating immune pathways with precision and for delivering therapeutics to targeted areas. Unlike traditional immunotherapies, aptamers can be designed to act as ICIs, co-stimulatory receptor stimulators, ligands for immune cells that inhibit tumor cells, or cytokine modulators, resulting in potent tumor-killing immune responses with lower systemic toxicity (Anantha Rajah et al., 2025; Jiang T. et al., 2025). They are also small, have high target specificity and low immunogenicity, and are amenable to multivalent and multifunctional design, enabling the development of bispecific constructs and combination strategies to overcome tumor-induced immunosuppression and enhance therapeutic efficacy. However, recent developments in chemical engineering, nucleic acid nanotechnology, and the optimization of aptamers by AI have helped translate these platforms from proof-of-concept studies to clinically-relevant immunotherapeutic applications (Tan et al., 2025). The subsequent sections detail the main engineering methods used for cancer immunotherapy with aptamers, including targeting immune checkpoints, activation of co-stimulatory receptors, aptamers specific to immune cells, and modulation of cytokines, along with their molecular mechanisms, pharmacological benefits, and novel applications in precision immuno-oncology.

### 6.1 Immune checkpoint targeting

Immune checkpoint molecules are key regulators of peripheral immune tolerance and are often exploited by tumors to evade immune surveillance and dampen the immune response against the tumor. Some of the best-studied checkpoints include PD-1, PD-L1, CTLA-4, T cell immunoglobulin and mucin domain-containing 3 (TIM-3), lymphocyte activation gene 3 (LAG-3), and T cell immunoreceptor with Ig and ITIM domains (TIGIT), which all contribute to T cell exhaustion, dysfunction, and resistance to immunotherapies (Figure 4) (Cillo et al., 2024; Guégan et al., 2024; Tang et al., 2025). Aptamers have unique pharmacological and engineering properties compared to the conventional antibody-based checkpoint blockade, due to small molecular size, high target affinity, chemical programmability, and the ability to interact with multiple or bispecific receptors, which may be useful for the penetration of tissue, local immune modulation, and the ability to be coupled with targeted delivery systems.

**FIGURE 4:**
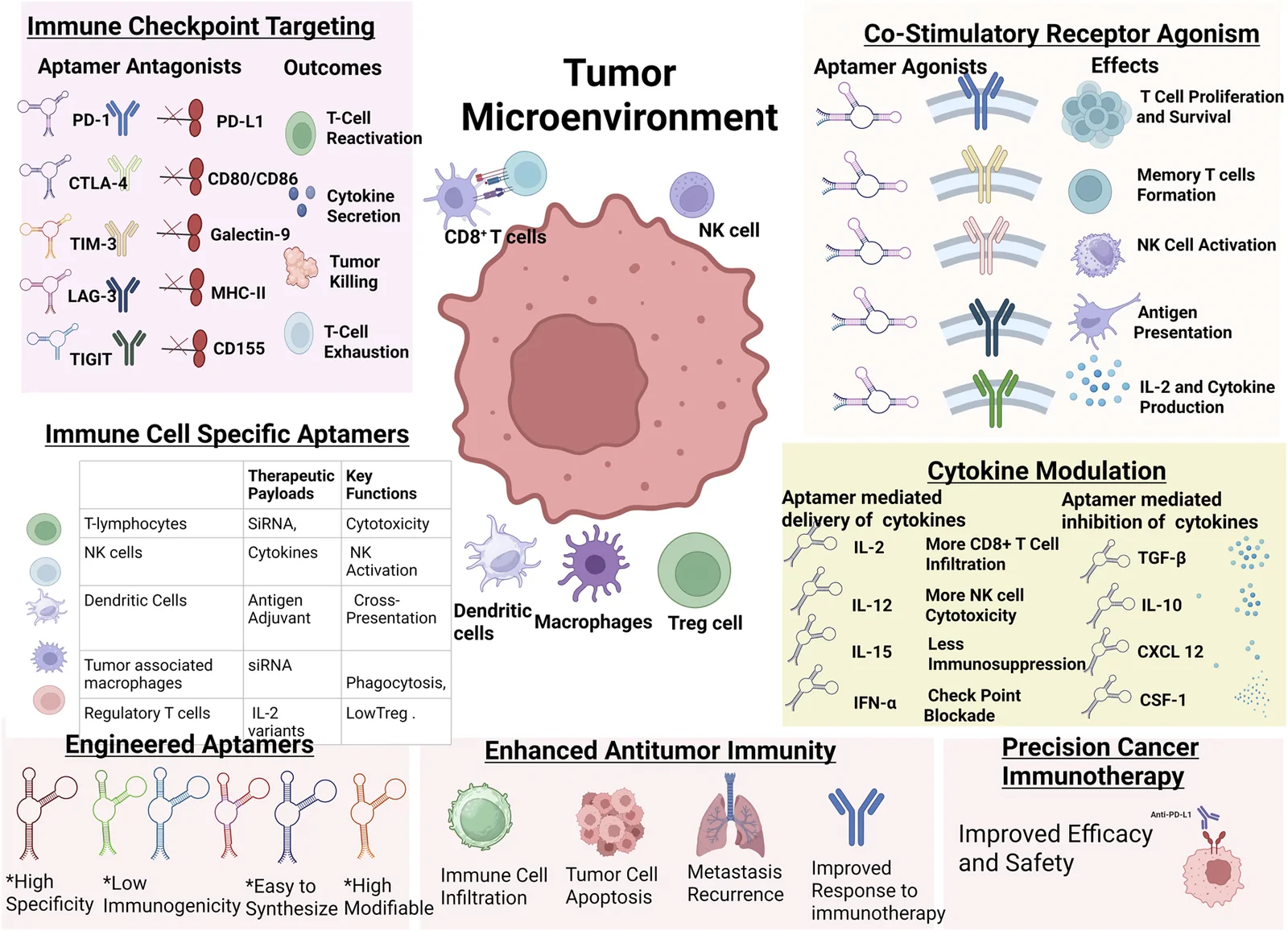
Engineering strategies of aptamers for precision cancer immunotherapy.

The small molecular size, high affinity, low immunogenicity, rapid tissue penetration, and ease of chemical engineering of aptamers are attractive features of these RNA molecules. Therapeutic aptamers targeting PD-1/PD-L1, CTLA-4, TIM-3, LAG-3, and TIGIT signaling have been developed to restore T cell activation, increase cytokine production, improve tumor killing by cytotoxic lymphocytes, and reverse immunosuppression in the TME (Guo et al., 2025; Kejamurthy et al., 2025). Advances in recent years have extended the use of immunotherapeutic single-target aptamers to multifunctional devices targeting multiple immune pathways. In pre-clinical models, bispecific and multivalent aptamers targeting combinations of checkpoints, such as PD-1/CTLA-4, PD-L1/TIGIT, or PD-L1/LAG-3, have been shown to be more effective at inhibiting adaptive immune resistance and compensatory checkpoint signaling, thereby enhancing tumor cell killing (Figure 4) (Abdu et al., 2025; Anantha Rajah et al., 2025). Aptamer-functionalized nanoparticles, exosomes, and nucleic acid nanostructures have also been used to selectively deliver checkpoint inhibitors alongside chemotherapeutics, siRNAs, miRNAs, cytokines, or CRISPR-based cargos, resulting in synergy in both the tumor cells and the immune microenvironment (Guo et al., 2022; Liao et al., 2026). However, there is growing evidence that aptamers targeting tumor-associated checkpoints can enhance the efficacy of adoptive cell therapies, cancer vaccines, and radiotherapy, and reduce systemic immune-related toxicities by specifically modulating the immune system in tumor tissues (Gao et al., 2025). Overall, these developments make aptamers promising next-generation immunotherapeutic tools for targeting immune checkpoints in precision cancer immunotherapy and personalized oncology.

### 6.2 Co-stimulatory receptor agonists

The generation of powerful and persistent anti-tumor immune responses requires the expression of co-stimulatory receptors, which are largely members of the tumor necrosis factor receptor superfamily (TNFRSF) and the CD28 receptor family. Some of the most therapeutically relevant are OX40 (CD134), 4-1BB (CD137), CD28, and CD40, which control the activation, proliferation, survival, and differentiation of T cells and antigen-presenting cells (Guercio et al., 2020; Yu L. et al., 2025). The activation of these receptors is a secondary signal necessary for optimal T cell priming following T cell receptor (TCR) engagement, leading to increased clonal expansion, cytokine production, cytotoxic function, and long-term immunological memory. In addition to agonistic monoclonal antibodies, aptamers are an alternative platform that can be designed as multivalent or dimeric molecules to cluster receptors and engineered to include tumor-targeting elements or therapeutic payloads (Figure 4) (McNamara et al., 2008; Pratico et al., 2013). Agonistic aptamers that target OX40, 4-1BB, CD28, and CD40 have been shown to strongly activate CD8^+^ and CD4^+^ T cells, DCs, and NK cells, leading to a superior, long-lasting antitumor immune response with minimal systemic activation (Pastor et al., 2013; Jiang T. et al., 2025). In recent years, a major challenge in the engineering of aptamers has been enhancing the therapeutic precision of co-stimulatory receptor agonists by engaging and activating cells in the tumor environment and by designing multifunctional constructs (Jiang T. et al., 2025; Liu et al., 2025c). The simultaneous binding of bispecific aptamers to both a TAAs and a co-stimulatory receptor can selectively activate the receptor in the TME, thereby avoiding systemic immune stimulation and reducing the risk of immune-related side effects associated with some agonistic antibodies (Figure 4) (Compte et al., 2018; Fu et al., 2023). Moreover, co-stimulatory aptamers are also beginning to be combined with ICB, cancer vaccines, and adoptive cell therapy, cytokine delivery, and nanomedicine-based platforms to elicit synergistic antitumor effects. Aptamer-mediated activation of OX40, 4-1BB, CD28, and CD40 is known to increase effector T cell infiltration, produce IFN-γ and IL-2, enhance DC maturation and antigen presentation, and promote long-lived memory T cells (Pastor et al., 2011; Jiang T. et al., 2025). These properties render agonistic aptamers as an attractive next-generation approach towards selectively boosting antitumor immunity and enhancing the effectiveness of precision cancer immunotherapy.

### 6.3 Immune cell-specific aptamers

Immunomodulatory aptamers have proven to be powerful tools for selectively targeting and regulating various immune cells in the TME. These aptamers can specifically bind to cell-surface receptors expressed on T lymphocytes, NK cells, DCs, macrophages, and regulatory T cells (Tregs), thereby enhancing therapeutic efficacy while reducing off-target immune activation (Chen et al., 2024b; Azhar et al., 2026). Aptamers against CD8^+^ T cells and activated T lymphocytes have been designed to transport immune-stimulating molecules, cytokines, siRNA, and components of the CRISPR system to increase the cytotoxic function, proliferation, and memory of T lymphocytes (Berezhnoy et al., 2014; Wen N. et al., 2025). Likewise, the use of aptamers that bind to NK cell receptors such as NKp30, NKp46, and NKG2D has shown promise in boosting the innate cytotoxicity against tumor cells. Targeting DCs with aptamers, particularly to the receptors DEC-205 (CD205), Clec9A, and CD11c, enhances their ability to take up antigen, cross-present it, and prime T cells, which is beneficial for cancer vaccines (Figure 4) (Wengerter et al., 2014; Zheng et al., 2023; Tan et al., 2025). These methods collectively leverage the specificity and modularity of aptamers to selectively activate antitumor immune responses while minimizing systemic immunotoxicity.

In recent years, the scope of immune cell-targeting aptamers has been broadened to selectively reprogram immunosuppressive cell populations in the TME. TAMs are being targeted with aptamers to block pro-tumorigenic signaling or shift TAMs from the immunosuppressive M2 phenotype to the pro-inflammatory M1 phenotype, thereby boosting antigen presentation, cytokine production, and recruitment of cytotoxic lymphocytes (Sylvestre et al., 2020; Qian et al., 2022; Yang Q. et al., 2024). At the same time, molecules that target Tregs through receptors like CD25, CCR4, GITR, immune checkpoint-associated receptors, and others are being studied to reduce Treg-mediated immune suppression and re-establish the function of effector T cells in the tumor (Wei X. et al., 2024; Abdelhamed et al., 2025). In parallel, the use of immune cell-specific aptamers in multifunctional nanoplatforms and bispecific constructs that target immune cells as well as TAAs enables local targeting of immune cells and synergistic combination with ICIs, adoptive cell therapies, and cancer vaccines. By coordinating innate and adaptive immune responses against malignant cells, these innovations establish a platform of aptamers that leverages immune cells as a versatile and highly promising approach to precision cancer immunotherapy (Li et al., 2019; Zheng et al., 2022; Chen et al., 2024b).

### 6.4 Cytokine modulation

In addition to their roles in coordinating the activation, proliferation, differentiation, and effector functions of both innate and adaptive immune cells, cytokines are major regulators of antitumor immunity. However, cytokines are often limited by dose-limiting toxicities, short circulating half-lives, and non-specific activation of the immune system, making them less useful clinically (Santollani et al., 2024; Beffinger et al., 2025). Aptamer engineering represents a highly selective approach for regulating cytokine signaling via targeted delivery to tumors, blocking immunosuppressive cytokine signaling pathways, or boosting local immune activation. Aptamers have been studied as a means of targeting immunostimulatory cytokines, including IL-2, IL-12, and IL-15, to selectively activate and expand CD8^+^ T cells, NK cells, and DCs, thereby reducing systemic exposure (Liu et al., 2017; Rajagopalan et al., 2017). In contrast, aptamers that target immunosuppressive mediators such as TGF-β, IL-10, CXCL12, and the CSF-1/CSF1R axis have been shown to reverse immunosuppression, increase immune cell infiltration, and enhance the response to checkpoint blockade in pre-clinical cancer models (Figure 4) (Giordano et al., 2024; Liu et al., 2025c).

Newly developed cytokine-modulating aptamers have emerged as versatile platforms for immunotherapeutic agents for remodeling the tumor immune microenvironment. Recently, bispecific aptamers and aptamer-modified nanoparticles have been increasingly developed for the co-delivery of cytokines with ICIs, siRNA, miRNA, chemotherapeutic agents, or even the CRISPR-based genome-editing system to achieve a synergistic antitumor effect (Zheng et al., 2022; Chen et al., 2024b). Moreover, stimuli-responsive delivery systems, which release cytokines in response to tumor-associated conditions such as acidic pH, elevated reactive oxygen species, or protease activity, further improve therapeutic precision and minimize immune-related adverse events (Yang C. et al., 2025; Feng et al., 2026). Recent strategies are also combining the effects of cytokines with agonism of co-stimulatory receptors and immune cell-specific targeting to simultaneously increase T cell activation, polarize macrophages toward the M1 phenotype, and enhance antigen presentation by DCs (Hogg et al., 2025). Together, these strategies position the cytokine-modulating aptamers as a significant addition to next-generation precision immunotherapy and enable selective activation of the immune system with a higher therapeutic index than current cytokine-based therapies (Qian et al., 2022; Chen et al., 2024b; Yang Q. et al., 2024).

### 6.5 Comprehensive overview of aptamer-based immune modulation, its potential, and limitations

Although immune modulation with aptamers has great promise, several pharmaceutical and translational hurdles currently limit their clinical use. The first is that aptamers are relatively small, which could lead to rapid renal clearance and a short half-life in the systemic circulation (Sun et al., 2025). The stability and blood circulation can be enhanced by chemical modification (e.g., PEGylation, introduction of modified nucleotides, binding to albumins, or formulation of nanoparticles), but this can affect tissue distribution, accessibility to cell targets, or biological activity (Yang S. et al., 2025). Second, the performance of the aptamers may be greatly affected by their susceptibility to nucleolytic degradation, inherent target heterogeneity, conformational instability, and variations in *in vitro* selection conditions and the physiological environment of the tumor (Zhou Y. et al., 2025). Such factors may result in resistance to target engagement and therapeutic activity *in vivo*, even if high affinity is observed under experimental conditions.

The third limitation is the less rigorous clinical evidence for the efficacy of immune-modulating aptamers, compared with the proven clinical efficacy of monoclonal antibodies and other immunotherapeutic approaches (Giordano et al., 2024). While aptamers can be designed to act as inhibitors of immune checkpoints, activators of co-stimulatory receptors, ligands for immune cells, or cytokine modulators, their pharmacokinetics and biodistribution, their immunological activity, dose–response relationships, and long-term safety demands further evaluation in clinically relevant models and human testing (Carmi Levy et al., 2024). Furthermore, multivalent, bispecific, and aptamer–nanoparticle constructs add complexity to functional properties and can pose additional challenges for manufacturing reproducibility, batch-to-batch quality control, formulation stability, and regulatory characterization (Kawamoto et al., 2024). Systematic assessment of potential off-target interactions, immune activation and suppression, and changes in activity after chemical modification is also needed.

The fourth and fifth essential biological barriers are the tumor microenvironment and the systemic immune system, respectively. Heterogeneous accumulation and pharmacological effects may result from interpatient and intratumoral variation in receptor distribution, ECM composition, vascular permeability, nuclease activity, and immune cell distribution (Thomas et al., 2024b; Doherty et al., 2025; Liu et al., 2025c). Moreover, aptamers can serve as molecular recognition elements and therapeutic delivery agents, incorporating multiple targeting ligands and therapeutic cargoes may reduce folding efficiency, aptamer stability, or the feasibility of scale-up manufacturing (Kawamoto et al., 2024). Therefore, the therapeutic benefits of aptamers do not mean that they are superior to antibody-, nanoparticle-, cytokine-, or gene-delivery-based immune-regulation approaches in general. Instead, aptamers offer an alternative, highly programmable platform with the key translational opportunity of combining molecular recognition with receptor modulation and targeted delivery, yet with the pharmacokinetics, stability, delivery, manufacturing, safety, and clinical validation challenges still to be overcome (Su et al., 2025).

## 7 Aptamer-based combination immunotherapy

While engineered aptamers have already demonstrated significant therapeutic value as single-agent immunomodulators, a growing body of evidence suggests that their full therapeutic potential lies in combination with other agents targeting complementary aspects of tumor growth and immune escape. Due to their specificity, favorable safety profiles, and ability to serve dual functions as therapeutic agents and delivery vehicles, aptamers may be combined with existing and new cancer treatments to enhance efficacy against the tumor and reduce side effects elsewhere in the body. Rational combinations allow modulation of tumor cells, immune checkpoints, TME, and intracellular oncogenic pathways to overcome treatment resistance and enhance the longevity of immune responses. Recent pre-clinical and early clinical research has underscored the potential of aptamer-based combination therapies with ICIs, classic chemotherapy, radiotherapy, adoptive cell therapy, cancer vaccines, and nanomedicine-based delivery systems to achieve more complete tumor regression, more infiltration of immune cells, and improved treatment outcomes (Giordano et al., 2024; Gao et al., 2025). These combination strategies are explained in the following sections, focusing on their mechanisms, therapeutic benefits, and the opportunities for translation to precision oncology and next-generation cancer immunotherapy.

### 7.1 Aptamers and ICIs

The pairing of aptamers with ICIs has proven a promising approach for improving intratumoral immune responses and overcoming the drawbacks of antibody-based checkpoint blockade therapy. Monoclonal antibodies against PD-1/PD-L1, CTLA-4, LAG-3, and, more recently, TIGIT have revolutionized cancer treatment; however, some patients remain resistant, some tumors do not respond, resistance develops over time, and tumors may not penetrate deeply enough into the body (Table 6) (Ryu et al., 2024; Abdu et al., 2025). While these monoclonal antibodies have revolutionized the treatment of cancer, some patients and some tumors are resistant to treatment, resistance develops over time, and, in some tumors, the antibodies do not penetrate deeply enough into the body (Di Giacomo et al., 2024; Malhotra et al., 2024). Aptamers offer an alternative strategy, as they have high target specificity, are not immunogenic, have excellent tissue penetration, and can be easily engineered into multivalent or bispecific forms. Therapeutic aptamers have direct anti-checkpoint activity or can selectively target CD8^+^ T cells in the TME to restore their function, reduce CD8^+^ T cell exhaustion, and increase tumor cell elimination with minimal activation of unwanted immune responses (Chen et al., 2024b; Wang Y. et al., 2024).

**TABLE 6:** Combination strategies integrating aptamers with contemporary cancer therapies.

Combination strategy	Primary mechanism of synergy	Major therapeutic advantages	Current stage of development/representative applications	References
ICIs	Coordinated reversal of T cell exhaustion and enhancement of immune activation	Improved response rates, greater immune specificity, potential reduction in systemic immune toxicity through localized targeting	Advanced pre-clinical studies; early clinical evaluation of aptamer-based checkpoint modulators	(Gao et al., 2025; Kejamurthy et al., 2025)
Chemotherapy	Tumor-selective delivery of cytotoxic agents and sensitization of resistant tumor cells	Higher intratumoral drug concentration, reduced off-target toxicity, induction of ICD	Extensive pre-clinical validation; ApDCs and targeted nanomedicine platforms	(Wen et al., 2024; Zhu et al., 2025)
Radiotherapy	Enhanced radiosensitization and modulation of DNA damage responses	Improved local tumor control, enhanced antitumor immune priming, lower radiation exposure to normal tissues	Pre-clinical studies using aptamer-guided radiosensitizers and radionuclide delivery systems	(Ren et al., 2025; Kan et al., 2026)
Photodynamic and photothermal therapy	Targeted light-activated tumor ablation with simultaneous immune activation	Precise spatial control, stimulation of systemic antitumor immunity, reduced collateral tissue injury	Rapidly expanding pre-clinical research involving photoresponsive nanoplatforms	(Wang et al., 2025c; Xu et al., 2025b)
OVs	Improved tumor-specific viral delivery and amplification of virus-induced immune responses	Enhanced viral localization, increased intratumoral immune activation, improved therapeutic selectivity	Early pre-clinical development integrating aptamers with engineered OVs	(Dymova et al., 2024a ; 2024b)
Cancer vaccines	Targeted antigen delivery and enhanced antigen presentation by DCs	Stronger cytotoxic T cell responses, improved immune memory, greater vaccine precision	Pre-clinical development with increasing interest in mRNA and personalized neoantigen vaccine platforms	(Zheng et al., 2023; Liu et al., 2026b)

Recently, the research emphasis has shifted toward developing a multifunctional aptamer-based platform that can be combined with ICBs to modify the tumor immune microenvironment (Camorani et al., 2020; Zheng et al., 2022; Chen et al., 2024b). Aptamer-functionalized nanoparticles, exosomes, and DNA nanostructures have been engineered for the coordinated delivery of ICIs alongside chemotherapeutics, RNA therapeutics, stimulator of interferon genes (STING) agonists, and/or CRISPR-based gene-editing systems, thereby ensuring the coordinated activation of innate and adaptive immunity (Zhang J. et al., 2023; Chen B. et al., 2024; Jiang et al., 2024). Furthermore, bispecific aptamers can target both immune checkpoints and TAAs, focusing the therapeutic activity of checkpoint blockade in tumor tissues, thereby enhancing therapeutic efficacy whilst minimizing systemic toxicity (Sun et al., 2022; Fu et al., 2023). Clinically, these combination regimens have been shown to promote DC activation, enhance infiltration and survival of CD8^+^ T cells and NK cells, decrease immunosuppressive cell subsets such as Tregs and M2-like TAMs, and improve response to checkpoint blockade in tumors (Qian et al., 2022; Chen et al., 2024b). Aptamer–ICI combinations are therefore a very promising strategy in precision oncology to tackle immune resistance and to obtain more durable clinical responses (Figure 5) (Zheng et al., 2022).

**FIGURE 5:**
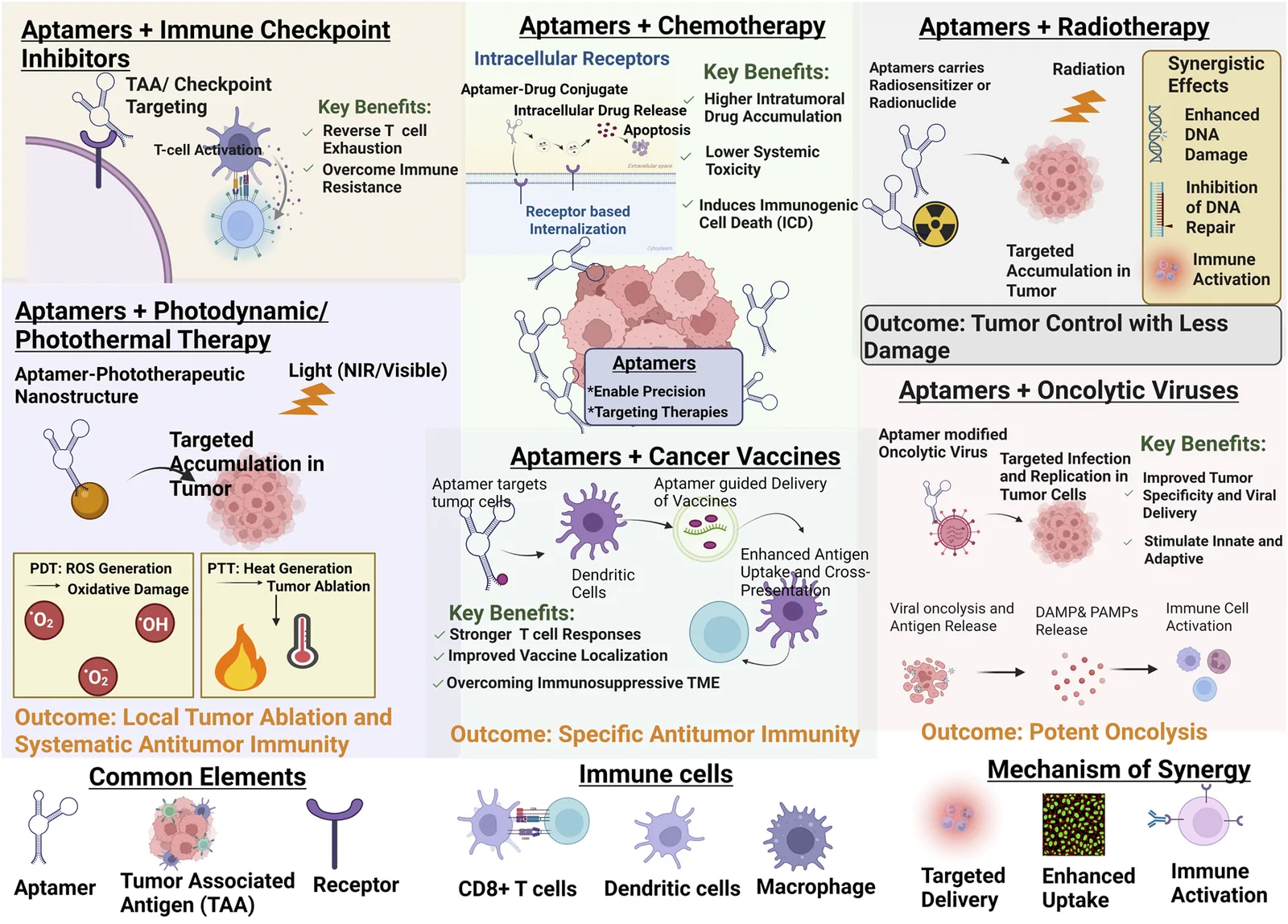
Aptamer-based combination therapeutic strategies for precision cancer immunotherapy.

### 7.2 Aptamers with chemotherapy

Aptamers combined with conventional chemotherapy have become a promising approach to increase the therapeutic index of cytotoxic drugs by increasing tumor targeting and reducing systemic toxicity. Although doxorubicin, paclitaxel, cisplatin, gemcitabine, oxaliplatin, camptothecin derivatives, and other chemotherapeutics have proven effective in clinical practice, their distribution is non-specific, leading to dose-limiting toxicities, multidrug resistance, and low drug accumulation in tumors (Yang W. et al., 2024; Wang et al., 2026b). The selective binding of aptamers to tumor-associated biomarkers, such as EpCAM, HER2, EGFR, PSMA, CD44, MUC1, and nucleolin, enables receptor-mediated uptake of chemotherapeutic agents by tumor cells, thereby overcoming these limitations (Xiao et al., 2024; Su et al., 2025). ApDCs and aptamer-functionalized nanocarriers can facilitate the controlled intracellular release of drugs, e.g., in response to TME pH, elevated glutathione concentrations, reactive oxygen species, or lysosomal enzyme activity, thereby enhancing intratumoral drug retention and minimizing off-target exposure (Wu Y. et al., 2025). This precision-targeting strategy would boost the efficacy of chemotherapy without affecting healthy tissues and improve its safety.

Recent advances have made aptamer-based chemotherapy (ACT) an emerging multifunctional therapeutic platform capable of simultaneously addressing drug resistance and boosting antitumor immunity (Kong et al., 2025; Zhu et al., 2025). Lipid nanoparticles that can be decorated with aptamers, polymeric nanoparticles, liposomes, exosomes, and DNA nanostructures are engineered to deliver chemotherapeutic drugs together with siRNAs, miRNAs, immune modulators, or ICIs in order to target multiple resistance pathways in the TME (Kim et al., 2022; Ren et al., 2024). Furthermore, aptamer-mediated delivery has been demonstrated to increase the accumulation of drugs in the cell, to inhibit drug efflux systems that require ATP binding (ABC transporters), to reduce the population of CSCs and to induce immunogenic cell death (ICD) including the exposure of calreticulin, the ATP release and the high mobility group box 1 (HMGB1) release, which are associated with DC activation and the priming of cytotoxic T cells (Yin et al., 2022; Jia et al., 2025). Such synergistic effects may enhance chemotherapy responsiveness, reduce tumor recurrence, and provide a strong rationale for combining aptamer-guided chemotherapy with targeted therapies and immunotherapy in precision oncology (Figure 5).

### 7.3 Aptamers with radiotherapy

Aptamers are being paired with radiotherapy to improve the accuracy of ionizing radiation therapy, increase tumor radiosensitivity, and reduce damage to normal tissues (Chen B. et al., 2024; Kan et al., 2025). Although radiotherapy is still an important treatment modality for cancer, it is often hindered by intrinsic or acquired radioresistance, tumor hypoxia, and radiation-induced toxicity. Aptamers have a highly selective targeting platform, with the ability to preferentially bind tumor-associated biomarkers, e.g., EGFR, HER2, PSMA, EpCAM, nucleolin, and integrin αvβ3; this enables the preferential accumulation of radiosensitizers or therapeutic radionuclides within malignant tissue (Table 6) (Bemidinezhad et al., 2025; Ren et al., 2025). Besides, aptamers can be attached to nanoparticles, gold nanostructures, and radionuclide-chelating systems to increase local radiation dose deposition and increase imaging and theranostic sensitivity in the tumor. This special approach enhances the effect of radiation while reducing irradiation of normal tissue, thereby improving the therapeutic approach (Ren et al., 2025; Kan et al., 2026).

In recent years, the use of aptamer-based targeting for radiosensitization has evolved into approaches that target the tumor immune microenvironment and DNA damage pathways. Aptamer-directed delivery of siRNAs, miRNAs, inhibitors of DNA repair, and immune modulators has been studied to block key mediators of radioresistance, such as DNA-dependent protein kinase, ATM, ATR, PARP, hypoxia-inducible factor 1-alpha, and EGFR-associated signaling, thereby inhibiting DNA repair and enhancing radiation-induced apoptosis (Ni et al., 2015; Zhang et al., 2018). Moreover, radiotherapy-induced ICD releases TAAs, ATP, HMGB1, and calreticulin, providing opportunities for synergistic combination therapy with aptamer-mediated ICB and cytokine modulation (Ren et al., 2024; He et al., 2025). Multifunctional nanoplatforms, which combine aptamers with radiosensitizers, immunotherapeutic agents, and imaging probes, are therefore emerging as a means to simultaneously improve tumor targeting, prime antitumor immunity, and facilitate image-guided precision radiotherapy (He et al., 2025; Ren et al., 2025). These developments make aptamer-assisted radiotherapy a promising part of next-generation multimodal cancer therapy (Figure 5).

### 7.4 Aptamers with photodynamic and photothermal therapy (PDT/PTT)

Aptamers coupled with PDT or PTT have become a very promising approach to selectively ablate tumors without causing significant damage to normal tissue. In PDT, radiation at these specific wavelengths activates the photosensitizer, generating reactive oxygen species that destroy tumor cells. In contrast, in PTT, near-infrared (NIR) light is used to heat the tumor cells, thereby causing irreversible cell death (Table 6) (Chen Y. et al., 2025; Negi et al., 2025). The clinical efficacy of both approaches is, however, often compromised by non-specific accumulation of photosensitizers or photothermal agents, insufficient penetration into the tumors, and off-target phototoxicity. Aptamers lack these drawbacks and selectively bind to tumor-associated markers such as EpCAM, HER2, EGFR, nucleolin, PSMA, CD44, and MUC1, thereby enabling receptor-mediated accumulation of photosensitizers or photothermal nanomaterials in tumor tissue (Chen Y. et al., 2025; Weng et al., 2026). Conventional PDT and PTT approaches have been enhanced by employing aptamer-functionalized gold nanoparticles, gold nanorods, graphene oxide, carbon nanomaterials, and semiconductor nanoplatforms, which have demonstrated improved tumor targeting, enhanced light-triggered therapeutic efficacy, and reduced systemic toxicity (Wang Z. et al., 2025; Xu H. et al., 2025).

Recent research has introduced new approaches that enable aptamer-guided PDT and PTT platforms to exert both direct tumor-killing and immunostimulatory effects. Light-induced death of tumor cells can trigger ICD by exposing calreticulin, releasing ATP, and releasing HMGB1, thereby initiating DC maturation, antigen presentation, and activation of CD8^+^ cytotoxic T lymphocytes (Li et al., 2024b; Weng et al., 2026). To this end, aptamer-based nanoplatforms are being developed to co-deliver ICIs, siRNAs, miRNAs, chemotherapeutic agents, cytokines, or STING agonists, thereby achieving synergistic phototherapy and immunotherapy effects (Li et al., 2025j; Chi et al., 2026). Moreover, stimuli-responsive systems that deliver therapeutic cargo upon irradiation with NIR light, acidic pH, and high intracellular glutathione levels further improve treatment precision and reduce off-target effects. In the field of precision oncology, these multifunctional platforms based on aptamer-guided photoimmunotherapy represent a promising strategy to achieve long-term systemic antitumor immunity, reduce metastatic progression, and overcome tumor heterogeneity (Figure 5) (Li S. et al., 2025; Tong et al., 2025).

### 7.5 Aptamers with oncolytic viruses (OVs)

The use of aptamers in combination with OVs is an emerging approach to enhance the tumor-targeting, therapeutic, and immunologic properties of virotherapy. OVs, such as genetically modified herpes simplex virus type 1, adenovirus, vaccinia virus, reovirus, measles virus, and vesicular stomatitis virus, selectively infect and lyse tumors, and activate antitumor immune responses via the release of TAAs and damage-associated molecular patterns (DAMPs) (Muharemagic et al., 2014; Dymova M. A. et al., 2024). However, they have been hampered by inefficient targeting, preexisting antiviral immunity, early systemic clearance, and limited intratumoral dissemination. They offer a promising alternative in the form of aptamers that bind tumor-associated markers such as EGFR, HER2, EpCAM, PSMA, CD44, and nucleolin, thereby enhancing viral targeting to the tumor without off-target viral infection (Table 6) (Muharemagic et al., 2014; Dymova et al., 2022). Aptamers may also be used to target receptors, increase viral uptake, and increase the therapeutic index of oncolytic virotherapy in viral delivery systems and/or multifunctional nanocarriers (Dymova M. A. et al., 2024).

Recent progress has focused on combining aptamer targeting with immuno-OVs to re-engineer the TME and enhance the systemic antitumor immune response (Zhao J. et al., 2025). Immunostimulatory molecules such as granulocyte-macrophage colony-stimulating factor, IL-12, IFN-β, or immune checkpoint blocking proteins can be engineered into OVs to boost tumor-specific viral replication, a strategy that can be complemented by aptamer-based targeting strategies to improve DC maturation, CD8^+^ T cell activation, and NK cell cytotoxicity (Chen L. et al., 2025; Xiao H. et al., 2025). Simultaneously, multifunctional platforms are developed to co-deliver OVs with siRNAs, miRNAs, immune checkpoint-targeting aptamers, cytokines, and/or chemotherapeutic agents to simultaneously suppress oncogenic signaling, overcome antiviral and immunosuppressive barriers, and improve intratumoral immune infiltration (Dharshini et al., 2025; Huang G. et al., 2025). While the majority of aptamer–OV technologies are still in pre-clinical development, advances in viral engineering, nucleic acid nanotechnology, and targeted delivery systems will likely accelerate the translation of precision cancer immunotherapy. These synergic approaches can enhance the safety, selectivity, and durability of oncolytic virotherapy and increase its clinical utility for a variety of solid and hematological cancers (Table 6; Figure 5) (Dymova M. A. et al., 2024; Hua et al., 2025; Xie et al., 2025).

### 7.6 Aptamers and cancer vaccines

Aptamers paired with cancer vaccines could prove a potent way forward in boosting the magnitude, specificity, and longevity of the on-target immune response against the tumor. Therapeutic cancer vaccines aim to induce tumor-specific T cell immunity via tumor-associated or neoantigens; however, their clinical efficacy is limited by inefficient antigen delivery, insufficient DC activation, poor lymph node trafficking, and an immunosuppressive TME (Zhou et al., 2024; Feng K. et al., 2025; Zhong et al., 2025). Aptamers have several distinct advantages as targeting ligands due to their high affinity, generally low inherent immunogenicity, and ease of chemical modification. Aptamers targeting receptors on professional antigen-presenting cells, including DEC-205 (CD205), Clec9A, CD11c, CD40, and XCR1, are used to deliver antigen to DCs selectively, thereby maximizing antigen uptake, cross-presentation via MHC-I, and activation of tumor-specific CD8^+^ cytotoxic T lymphocytes (Wengerter et al., 2014; Clark et al., 2024; Gomase et al., 2025; Wang et al., 2026b). Moreover, aptamers that bind to tumor-associated biomarkers can help to localize vaccines in the TME, enhancing the precision and efficacy of therapeutic vaccination.

In recent years, significant progress has been made in developing aptamer-based cancer vaccines as multifunctional immunotherapeutic platforms that integrate antigen delivery with immune modulation. Aptamers are being used in an increasing number of lipid nanoparticles, polymeric nanoparticles, DNA nanostructures, and self-assembling nucleic acid scaffolds to deliver neoantigens, mRNA, DNA vaccines, STING agonists, TLR agonists, cytokines, or ICIs, thereby creating coordinated innate and adaptive immune responses (Dong Y. et al., 2024; Guo et al., 2024; Wang et al., 2026b). These combination platforms induce DC maturation, boost T cell priming, expand long-lived memory T cell populations, and reduce tumor-induced immunosuppression, thereby enhancing the efficacy of vaccination against primary and metastatic tumors. Furthermore, the use of aptamers in combination with personalized neoantigen vaccines and mRNA-based immunization strategies is attracting considerable interest as a precision oncology strategy with the potential to tailor immune responses to individual tumors based on their molecular features (Figure 5) (Wang et al., 2026b).

### 7.7 Critical comparison of aptamer-based combination of therapeutic strategies

The therapeutic advantages of aptamer-based combination strategies are accompanied by modality-specific limitations that should be considered when interpreting their translational potential. Combination with ICIs can simultaneously improve tumor targeting and restore antitumor T-cell activity, but efficacy may still depend on the tumor’s pre-existing immune contexture, and excessive immune activation may increase the risk of immune-related toxicity (Ren et al., 2023; Altan et al., 2024). Aptamer-guided chemotherapy can increase tumor-localized drug exposure and potentially reduce systemic toxicity, but therapeutic benefit remains dependent on target expression, receptor-mediated internalization, intracellular drug release, and the pharmacology of the cytotoxic payload; heterogeneous or heterogeneous/low target expression can therefore limit efficacy (Su et al., 2025; Liu Y. et al., 2026). Aptamer-assisted radiotherapy can improve tumor localization and radiosensitization and may enhance ICD, but normal-tissue radiation exposure, tumor hypoxia, limited penetration into bulky tumors, and cumulative radiation toxicity remain important constraints (Kan et al., 2026). Similarly, aptamer-guided photodynamic or photothermal therapy provides spatially controlled tumor ablation and can stimulate antitumor immunity, but its effectiveness is constrained by light penetration, tumor depth, photosensitizer or nanomaterial distribution, and, for photodynamic therapy, oxygen availability (Weng et al., 2026).

Aptamer-targeted OVs offer the possibility of combining selective tumor infection with immunogenic tumor lysis and systemic immune activation; however, antiviral immunity, variable receptor expression, inefficient intratumoral dissemination, viral safety, and the complexity of manufacturing engineered viral platforms remain major barriers (Dymova M. A. et al., 2024; Fu et al., 2026). Aptamer–cancer vaccine combinations can improve antigen delivery to DCs, cross-presentation, and tumor-specific T-cell priming, but vaccine efficacy may be limited by antigen heterogeneity, inadequate antigen presentation, immunosuppressive tumor microenvironments, and the need to generate sufficiently strong and durable immune memory (Zheng et al., 2023; Wang et al., 2026b). More broadly, combining aptamers with multiple therapeutic modalities can increase mechanistic complementarity but also increases construct complexity, manufacturing and quality-control requirements, pharmacokinetic and pharmacodynamic uncertainty, potential drug–drug or platform–platform interactions, and the difficulty of attributing efficacy or toxicity to individual components (Chi et al., 2026). In addition, aptamer-specific limitations—including nuclease degradation, renal clearance, target heterogeneity, off-target interactions, and variable tumor penetration—are not necessarily eliminated by combination therapy and may become more consequential as construct complexity increases (Doherty et al., 2025; Liu et al., 2025c). Therefore, the selection of an aptamer-based combination may be guided by the biological mechanism of resistance being addressed, target expression, tumor accessibility, expected pharmacodynamic interactions, and the cumulative safety and manufacturing requirements of the complete therapeutic platform, rather than by the presence of synergy alone.

Therapeutic aptamers selectively bind to tumor-associated biomarkers (e.g., EGFR, HER2, EpCAM, PSMA, CD44, MUC1, nucleolin) or immune regulatory molecules, enabling targeted delivery of therapeutics and localized modulation of the TME. By inhibiting immune inhibitory pathways such as PD-1/PD-L1, CTLA-4, LAG-3, TIM-3, and TIGIT, aptamers can, in combination with ICIs, help restore T cell function and improve cytotoxic immune responses (Fu et al., 2023; Lai et al., 2023). Aptamer-guided chemotherapy selectively targets tumor cells, leading to enhanced uptake by tumor cells, the ability to overcome therapeutic resistance, and reduced systemic toxicity. The targeted delivery of radionuclides or radiosensitizers using aptamers as targeting agents, along with the induction of ICD and tumor immune activation, are the features that make aptamer-assisted radiotherapy a promising targeted radiosensitization approach (Wen et al., 2024; Li L. et al., 2025). Receptor-specific targeting of PDT/PT agents with aptamers enables their accumulation at the target site, leading to the generation of reactive oxygen species or local hyperthermia, respectively, which are responsible for tumor ablation and immune system activation (Table 6) (Li et al., 2025j; Zhang X. et al., 2025). OVs targeted by aptamers enhance the viral infection and replication in the tumor, leading to the release of tumor antigens, induction of innate and adaptive immune responses, and tumor lysis. In combination with cancer vaccines, aptamers can deliver antigen to DCs, stimulate antigen cross-presentation, and induce potent tumor-specific T cell responses and long-term immunologic memory (Zheng et al., 2023; Dymova M. A. et al., 2024). Together, these synergistic approaches can reshape the tumor immune microenvironment, enhance the specificity of therapeutic responses, overcome immune evasion and treatment resistance, and serve as a prototypical platform for novel precision oncology applications.

## 8 Conclusion

Aptamers have potential applications in combination drugs with ICIs, chemo, radiotherapy, PDT, PTT, OVs, and vaccines, with the goal of enhancing tumor-specific immune modulation and drug delivery to tumors with minimal systemic exposure, which may only be realized through extensive preclinical and clinical studies.

Improvements in SELEX, Cell-SELEX, *in vivo* SELEX, HTS, microfluidics, AI-based sequence optimization, and structural engineering have greatly increased the capacity to discover and enhance aptamers for clinically relevant tumor and immune-associated targets, as discussed throughout this review. They possess the potential to act as receptor agonists or antagonists, influence oncogenic and immune signaling pathways, and selectively target the tumor, tumor stem cells, tumor vasculature, and stromal components, offering a wide range of molecular mechanisms to support precision oncology applications.

Another significant advantage of aptamers is that they can be used as targeted cargo carriers for a wide range of therapeutics, including chemotherapeutics, siRNAs, miRNAs, proteins, peptides, and CRISPR systems. In particular, ApDCs, nucleic acid delivery systems, immune checkpoint-targeting aptamers, co-stimulatory receptor agonists, cytokine-modulating platforms, and immune cell-specific aptamers offer opportunities to simultaneously regulate both tumor cells and the tumor immune microenvironment. Aptamers also hold promise as part of multimodal combinations with ICIs, chemotherapy, radiotherapy, PDT, PTT, OVs, and vaccines to achieve and potentially improve selectivity and tumor-localized immune activation while reducing systemic exposure. Although these benefits require validation in appropriate *in vivo* and clinical studies. However, several pharmaceutical, biological, manufacturing, and regulatory challenges remain to be overcome before the clinical translation of aptamer-based cancer therapeutics is possible. These include rapid renal clearance and nuclease-mediated degradation; context-dependent stability and target engagement; heterogeneous target expression within tumors; variable biodistribution; limited characterization of the relationships between pharmacokinetics and pharmacodynamics; and potential off-target or unintended immunological effects. The production of reproducibility, formulation stability, scale-up, and regulatory characterization are further challenges that may be introduced with more complex bispecific, multivalent, and aptamer–nanomaterial constructs. Finally, future research and development should incorporate rational chemical modification, optimized delivery and half-life-extension approaches, thorough and systematic pharmacokinetic and toxicological evaluation, standardized manufacturing and quality-control processes, and carefully designed clinical trials to establish the safety, pharmacological activity, and therapeutic efficacy of aptamer-mediated immune modulation.

## Glossary

**2′-F**: 2′-fluoro
**2′-OMe**: 2′-O-methyl
**ABC**: ATP-Binding Cassette
**ACT**: Aptamer-Based Chemotherapy
**AI**: Artificial Intelligence
**ApDCs**: Aptamer–Drug Conjugates
**ATM**: Ataxia-Telangiectasia Mutated
**ATR**: Ataxia-Telangiectasia and Rad3-Related Protein
**CAFs**: Cancer-Associated Fibroblasts
**CSCs**: Cancer Stem Cells
**CD**: Cluster of Differentiation
**CRISPR**: Clustered Regularly Interspaced Short Palindromic Repeats
**CRISPRa**: CRISPR Activation
**CRISPRi**: CRISPR Interference
**CTLA-4**: Cytotoxic T-Lymphocyte-Associated protein-4
**DCs**: Dendritic Cells
**DAMPs**: Damage-Associated Molecular Patterns
**EGFR**: Epidermal Growth Factor Receptor
**EpCAM**: Epithelial Cell Adhesion Molecule
**ECM**: Extracellular Matrix
**HER2**: Human Epidermal Growth Factor Receptor 2
**HTS**: High Throughput Sequencing
HMGB1: High-Mobility Group Box 1
**ICB**: Immune Checkpoint Blockade
**ICD**: Immunogenic Cell Death
**ICIs**: Immune Checkpoint Inhibitors
**KRAS**: Kirsten Rat Sarcoma viral oncogene homolog
**LAG-3**: Lymphocyte Activation Gene 3
**LNAs**: Locked Nucleic Acids
**MHC-I**: Major Histocompatibility Complex Class I
**miRNA**: MicroRNA
**mRNA**: Messenger RNA
**MUC1**: Mucin-1
**NIR**: Near-Infrared
**NK**: Natural Killer
**OVs**: Oncolytic Viruses
**PARP**: Poly(ADP-Ribose) Polymerase
**PD-1**: Programmed Cell Death Protein 1
**PD-L1**: Programmed Death-Ligand 1
**PSMA**: Prostate-Specific Membrane Antigen
**SELEX**: Systematic Evolution of Ligands by Exponential Enrichment
**siRNA**: Small Interfering RNA
**STING**: Stimulator of Interferon Genes
**TAA**: Tumor-Associated Antigen
**TAMs**: Tumor-Associated Macrophages
**TCR**: T Cell Receptor
**TGF-β**: Transforming Growth Factor-beta
**TIGIT**: T Cell Immunoreceptor with Ig and ITIM Domains
**TIM-3**: T Cell Immunoglobulin and Mucin Domain-Containing 3
**TNFRSF**: Tumor Necrosis Factor Receptor Superfamily
**Tregs**: Regulatory T Cells
**TME**: Tumor Microenvironment.
